# Targeting Reactive Oxygen Species and Inflammation in Sepsis-Induced Liver Injury with Naturally Derived Superoxide Dismutase-Mimicking Carbon Dots

**DOI:** 10.34133/bmr.0249

**Published:** 2025-09-05

**Authors:** Chonglei Zhong, Nannan Song, Ping Huang, Liwen Han, Jiguo Zhang, Zhiyuan Lu, Lei Wang

**Affiliations:** School of Pharmaceutical Sciences & Institute of Materia Medica, State Key Laboratory of Advanced Drug Delivery and Release Systems, Medical Science and Technology Innovation Center, Shandong First Medical University & Shandong Academy of Medical Sciences, Jinan 250117, China.

## Abstract

Sepsis-induced liver injury (SILI) is a serious complication of septicemia and contributes to high rates of patient death. SILI is characterized by excessive hepatic reactive oxygen species (ROS) generation, leading to inflammatory response activation and the release of inflammatory mediators that yield liver damage. Efforts to design drugs that can mitigate oxidative stress and inflammatory factor production are thus vital to protecting patients against SILI. Nevertheless, effective pharmacological interventions for SILI therapy are currently absent. Here, natural superoxide dismutase (SOD)-mimetic carbon dots (G-CDs), derived from the traditional medicine plant *Glycyrrhiza*, with robust ROS-scavenging activity were designed and synthesized as a novel treatment for SILI. These G-CDs possess abundant surface hydroxyl and carbonyl groups such that they can effectively mediate SOD-like enzyme activity exceeding 13,340 U/mg to alleviate ROS overproduction and associated inflammation. In a murine model of lipopolysaccharide-induced SILI, these G-CDs effectively reduced hepatic inflammation, oxidative injury, and tissue damage. From a mechanistic perspective, these G-CDs were found to preserve liver integrity through the activation of Keap1/Nrf2-mediated antioxidant signaling and the inhibition of NF-κB-dependent inflammation, thereby reducing the levels of hepatic inflammation and oxidative stress. In summary, these results highlight the promise of G-CDs as therapeutic candidates capable of treating SILI by mitigating oxidative stress-associated liver injury.

## Introduction

Sepsis is a complicated systemic inflammatory disease that can be triggered by various pathogens, including many viruses and bacteria, through the induction of an inadequately regulated host immune response to the underlying infection [1]. Lipopolysaccharide (LPS) is one of the primary components of the outer membrane of Gram-negative bacteria, and its interaction with immune cells can trigger the secretion of inflammatory factors and the initiation of a cascading inflammatory response that results in extensive immunological dysregulation [2]. The liver is the primary organ responsible for detoxification, and as a result, it is highly susceptible to invasion and infection early during sepsis [3]. Excessive levels of pro-inflammatory cytokines and ROS in the liver produce excessive inflammatory activity and oxidative stress, leading to sepsis-induced liver injury (SILI) [4]. Glucocorticoids are the main drugs used to treat SILI owing to their ability to limit inflammation and ROS production. However, the clinical utility of these drugs is hampered by the fact that high doses are required for satisfactory efficacy, poor water solubility, and the potential for substantial side effects including metabolic dysregulation and immunosuppression. Efforts to design new efficient drugs capable of clearing ROS and mitigating inflammation are thus vital to treating SILI.

Carbon dots (CDs) are carbon-based nanomaterials with zero-dimensional structures, typically less than 10 nm in size, and they exhibit excellent biocompatibility, suitable particle size, and tunable biological properties [5]. In comparison with CDs synthesized from chemical precursors, biomass-derived CDs have attracted marked attention due to their low cost, reduced biotoxicity, and distinctive biological activities [6,7]. Phytopharmaceuticals have been employed for millennia as both preventive and therapeutic agents against diseases [8], and they contain a wide array of bioactive compounds that offer a natural route for incorporating heteroatomic groups into the structure of CDs [9]. This makes phytomedicines indispensable precursors for synthesizing biomass-derived CDs with unique physicochemical and biological properties [10,11]. For example, ginger-derived CDs have shown the potential to induce apoptosis and inhibit tumor growth [12]. In addition, Lu et al. synthesized CDs from the herb *Codonopsis pilosula*, which exhibited erythropoietic activity by modulating the Janus kinase (JAK)/signal transducer and activator of transcription (STAT) pathway [13]. Nevertheless, the development of cost-effective and multifunctional CDs from natural plants with potent anti-inflammatory and antioxidant properties for the treatment of SILI remains a major challenge.

*Glycyrrhiza* is abundant in organic acids, flavonoids, and terpenoids, which possess pharmacological activities such as anti-inflammatory, antibacterial, hepatoprotective, detoxifying, and heat-clearing properties, and is extensively utilized in the treatment of inflammatory diseases [14]. Inspired by the traditional Chinese medicine practice of enhancing the pharmacological activity of *Glycyrrhiza* through high-temperature carbonization, we synthesized G-CDs for the first time using hydrothermal treatment in this study. These G-CDs were able to effectively eliminate elevated levels of intracellular ROS and protect mitochondrial function from the effects of oxidative stress through the activation of the Keap1/Nrf2 antioxidant signaling axis. In both zebrafish and mouse-based models of inflammation, these G-CDs also presented with robust anti-inflammatory effects that were related to the inhibition of nuclear factor κB (NF-κB)-dependent inflammation. These results provide promising opportunities to further explore new approaches to leveraging CDs derived from natural materials.

**Fig. 1.**
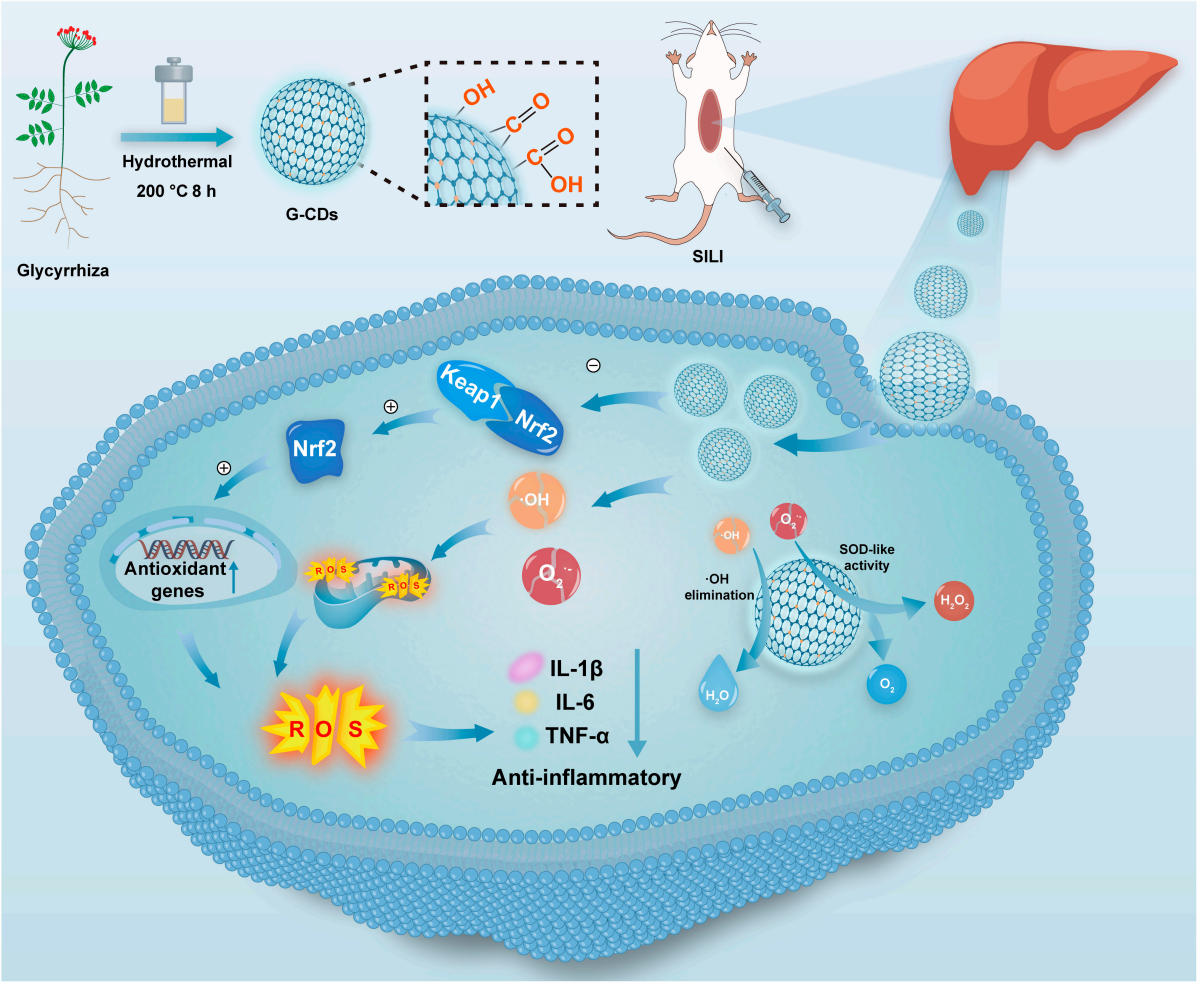
Schematic representation of the preparation of fluorescent G-CDs with high levels of SOD activity and treatment of SILI.

In summary, our study developed a multifunctional nanomedicine derived from medicinal plants. G-CDs demonstrated therapeutic efficacy against SILI through multiple molecular mechanisms, particularly via their SOD-like nanoenzyme activity (Fig. 1). This offers a promising strategy for the development of novel CD-based therapies for SILI.

## Materials and Methods

### Chemicals and reagents

*Glycyrrhiza* was obtained from BWT Chinese Herbal Medicine Drinks Slice (Shandong, China) and was authenticated by L.-W. Han (Shandong First Medical University). Nitrotetrazolium blue chloride (NBT), riboflavine, L-methionine, CuSO_4_, 3,3′,5,5′-tetamethylbenzidine dihydrochloride (TMB), ibuprofen, potassium bromide (KBr), FeSO_4_·7H_2_O, 1,3-propane-sultone (PS), triethylamine (TEA), 1,4 dioxane, and NaAc-Hac (pH 5.2) were from Rhawn (Shanghai China). A nitric oxide (NO) assay kit was from Nanjing Jiancheng Bioengineering Institute (Jiangsu, China), while TargetMol (Shanghai, China) was the source of the Cell Counting Kit-8 (CCK-8) kit. LPS from *Escherichia coli* 055:B5 and dimethyl sulfoxide (DMSO) were from Sigma-Aldrich (MO, USA). MitoSOX Red was from Thermo Scientific (MA, USA). 2',7'-Dichlorodihydrofluorescein diacetate (DCFH-DA) was from AmBeed (Shanghai, China). The utilized bicinchoninic acid (BCA) protein assay kit was from Beyotime (Shanghai, China). H_2_O_2_ (30%) was from HUSHI (Shanghai, China). Antibodies specific for IκB kinase β (IKKβ), IκBα, P65, NLRP3, cyclooxygenase-2 (COX-2), inducible nitric oxide synthase (iNOS), Keap1, Nrf2, HO-1, and glyceraldehyde-3-phosphate dehydrogenase (GAPDH) were from Proteintech (Wuhan, China), whereas antibodies specific for p-IKKβ, p-IκBα, and p-P65 were from Cell Signaling Technology (MA, USA). Primer sequences are listed in Table S1.

### G-CDs preparation and characterization

Roasted *Glycyrrhiza* fragments (5 g) were immersed in 60 ml of deionized water and heated for 8 h at 200 °C in an autoclave. The solution was then allowed to cool to room temperature before passing through a 0.22-μm microporous membrane, yielding a transparent brown solution that was dialyzed for 10 h with a 500-kDa dialysis membrane for G-CDs purification. The resultant G-CDs sample was then freeze-dried to produce a powder. A Talos F200S transmission electron microscope (TEM) (FEI, OR, USA) was used to assess G-CDs morphological and structural properties at an accelerating voltage of 200 kV. G-CDs thickness was additionally assessed with a Dimension Icon atomic force microscope (AFM) (Bruker, Karlsruhe, Germany). Fourier transform infrared spectroscopy (FTIR) in the 400 to 4,000 cm^−1^ range was also performed using a Thermo Scientific spectrophotometer (USA) to facilitate the characterization of G-CDs functional groups. X-ray photoelectron spectroscopy (XPS) was also performed with a K-α x-ray photoelectron spectrometer (Thermo Scientific, USA) to evaluate the chemical bonding and elemental composition of these G-CDs. An F-7000 fluorescence spectrometer (Hitachi, Japan) was used for fluorescence spectral analyses with a 450-nm excitation wavelength. A Rigaku D/MAX-2600 (Japan) with a scan rate of 2°/min was used to collect x-ray diffraction (XRD) data.

### Synthesis of G-CDs-A and G-CDs-B

Five milliliters of 10 mg/ml G-CDs, 1 g of PS, 20 ml of 1,4 dioxane, and 2 ml of TEA were placed in a beaker at 40 °C closed stirring for 24 h, and the solvent was removed by rotary evaporation. The product was redispersed in water and dialyzed in a 500-Da dialysis bag, 0.1 M NaCl solution at room temperature for 1 day, and ultrapure (UP) water for 3 d. The obtained product was stirred with 20 ml of 0.5 M NaOH at 40 °C for 24 h. The pH was adjusted to neutral with HCl, dialyzed in UP water for 3 d, and freeze-dried to obtain G-CDs-A. The preparation of G-CDs-B differed from that of G-CDs-A, as it required pretreatment before the reaction. Specifically, 40 mg of G-CDs was mixed with 50 ml of 0.5 M NaBH_4_ solution and stirred at room temperature for 12 h. Following this, the mixture was dialyzed in UP water for 3 d.

### G-CDs total antioxidant analyses

A T-AOC Assay Kit (Beyotime, China) was used as directed. The characteristic absorbance peak of ABTS^•+^ at 414 nm was monitored in response to different G-CDs concentrations within 60 s, enabling the detection of the remaining ABTS^•+^, obtaining the absorption spectra for visible light for each concentration group via continuous spectral scanning.

### Analyses of SOD-like activity

G-CDs SOD-like activity was quantified with a Total Superoxide Dismutase Assay Kit (Dojindo, Japan) as directed based on the inhibition of the competitive WST-1 [(2-(4-Iodophenyl)-3-(4-nitrophenyl)-5-(2,4-disulfophenyl)-2*H*-tetrazolium sodium salt] reaction. The O_2_^•−^ scavenging activity of G-CDs was measured via the NBT method. Briefly, G-CDs (0 to 800 μg/ml) and NBT (0.05 mM) were combined with L-methionine (13 mM) and riboflavin (20 μM) in 25 mM phosphate-buffered saline (PBS) (pH 7.4) followed by light-emitting diode (LED) irradiation for 3 min. The change in absorbance at 560 nm was then measured to assess O_2_^•−^ removal.

### Analyses of hydroxyl radical scavenging activity

The ability of G-CDs to scavenge hydroxyl radicals (•OH) was assessed with a modified version of a protocol published previously [15]. Briefly, 100 μl of G-CDs (0, 50, 100, 200, 400, and 800 μg/ml) was combined with 20 μl of FeSO4·7H_2_O [10 mM in deionized (DI) water], 65 μl of H_2_O_2_ (100 mM in DI water), 10 μl of NaAc-Hac buffer (pH 5.2), and 5 μl of TMB (10 mM in DMSO) for 1 h in the dark at room temperature. Absorbance at 652 nm was then analyzed to assess product formation, analyzing G-CD free radical scavenging activity as follows:

$$\text{Eliminate}\%=\left(A_{TMB}-A_{\text{Sample}}\right)/A_{TMB}\times 100\%$$ (1)

where *A* denotes the absorbance of the solution at 652 nm in the presence or absence of G-CDs.

### Intracellular ROS analyses

ROS levels in RAW264.7 cells were quantified with DCFH-DA (AmBeed, China) used as directed. Briefly, DCFH-DA was prepared in serum-free medium at 10 μM, followed by incubation for 30 min at 37 °C, washing the cells thrice with PBS to remove any free DCFH-DA. A fluorescence microscope was then used to detect intracellular ROS based on the intensity of green fluorescence.

### Mitochondrial membrane potential analyses

A JC-1 staining kit (MCE, Shang, China) was used as directed to quantify mitochondrial membrane potential (MMP). Briefly, serum-free medium containing 2 μM JC-1 was incubated with RAW264.7 cells for 20 min at 37 °C. These cells were then washed 2 times using PBS and imaged with a fluorescence microscope. In the resultant images, red and green fluorescence were respectively associated with polymeric and monomeric JC-1.

### Malondialdehyde and SOD analyses

After discarding culture supernatants and collecting cells via centrifugation, they were disrupted via sonication and centrifuged again at 12,000 rpm. The resultant supernatants were then analyzed with assay kits based on provided instructions, while a BCA assay was used to quantify protein levels in these cell samples.

### MitoSOX analyses

MitoSOX Red (Thermo) was used as directed to assess mitochondria superoxide production. Briefly, a 500 nM working solution was prepared and used to stain RAW264.7 cells for 30 min in a standard culture incubator. Cells were then washed thrice with PBS and imaged under a fluorescence microscope, with the intensity of the red fluorescence signal being proportional to the degree of mitochondrial superoxide production.

### RNA-sequencing and bioinformatics analyses

RAW264.7 cells were plated in 6-well plates with LPS (1 μg/ml) with or without G-CDs (400 μg/ml) for 6 h, after which TRIzol was used to extract total RNA from these cells and RNA extraction was conducted as previously described [16]. A NanoDrop spectrophotometer and a Bioanalyzer 2100 system were used to quantify RNA yields and assess RNA quality, after which an Illumina NovaSeq 6000 platform was used to assess changes in gene expression. The resultant data were used to select genes that were up-regulated or down-regulated that were relevant to oxidative stress and inflammation. As these data were from murine cells, while connectivity map (cMAP) data were from human cell lines, murine gene identifiers were converted to human gene identifiers with the HomoloGene database (https://www.ncbi.nlm.nih.gov/datasets/gene/), introducing the homologene into the cMAP website and performing analyses with the query tool. The RNA-sequencing (RNA-seq) data have been deposited in the National Center for Biotechnology Information (NCBI) Gene Expression Omnibus (GEO) repository with the accession GEO: GSE278841 (https://www.ncbi.nlm.nih.gov/geo/info/faq.html#holdprivate).

### In vivo evaluation

#### Anti-inflammatory effects of G-CDs in the CuSO₄-induced zebrafish model

At 72 h post-fertilization (hpf), zebrafish larvae with DsRED2-labeled neutrophils without embryonic membranes were added to 6-well plates (20 larvae/well) and separated into control, model, positive control (20 μM ibuprofen), and G-CDs (100, 200, and 400 μg/ml) treatment groups. The positive control and G-CDs groups were pretreated for 6 h, after which inflammation was initiated by adding 20 μM CuSO₄ to all wells other than the control wells for 2 h. The anti-inflammatory effects of G-CDs were quantified by the random selection of 6 zebrafish per group and by assessing neutrophil distributions using fluorescence microscopy.

#### Therapeutic effects of G-CDs in a sepsis-induced mouse model

Male BALB/c mice (7 to 8 weeks old, 18 to 20 g, Charles River, Beijing, China) were housed under specific pathogen-free controlled conditions (25 °C, 50% humidity) with free food and water access. These mice were randomized into control, LPS, LPS + dexamethasone (3 mg/kg), LPS + G-CDs (25 mg/kg), and LPS + G-CDs (50 mg/kg) groups. A murine model of SILI was established by administering a single dose of LPS (5 mg/kg) intraperitoneally for 24 h, with mice in the LPS + G-CDs group having been pretreated with G-CDs for 3 d and intraperitoneally treated using G-CDs (100 μl, 25 or 50 mg/kg) 60 min following LPS injection. Equal volumes of sterile PBS were administered to mice in the control and LPS groups. The positive control mice were administered 3 mg/kg of dexamethasone intraperitoneally. At 24 h after modeling, isoflurane was used to euthanize animals. Isoflurane was also used as an anesthetic to minimize pain and distress throughout the study. The Ethics Committee of the Experimental Animal Centre of Shandong First Medical University approved all animal studies (Shandong, China, no. W202410180679) and conforms to all the relevant guidelines and regulations.

#### Biosafety assessment of G-CDs

For biosafety assays, 6- to 8-week-old wild-type BALB/c mice were separated into 3 groups that were injected intraperitoneally with G-CDs (100 or 500 mg/kg) or saline control. The body weights of these animals were recorded for 7 d, after which blood samples were harvested and evaluated with a hematology analyzer. A liver and renal function activity assay kit was also used to analyze aspartate aminotransferase (AST), alanine aminotransferase (ALT), blood urea nitrogen (BUN), and creatinine (CREA) levels in these animals. Major organs (heart, lungs, kidneys, spleen, liver) from these mice were also subjected to histological examination following hematoxylin and eosin (H&E) staining.

### Statistical analysis

The statistical data were presented as means ± standard deviation (SD). Appropriate statistical tests, including *t* tests and analysis of variance (ANOVA), were employed to compare different groups. The values of *P* < 0.05, *P* < 0.01, and *P* < 0.001 indicated statistical difference, significant statistical difference, and extremely significant statistical difference, respectively.

## Results

### G-CDs characterization

**Fig. 2.**
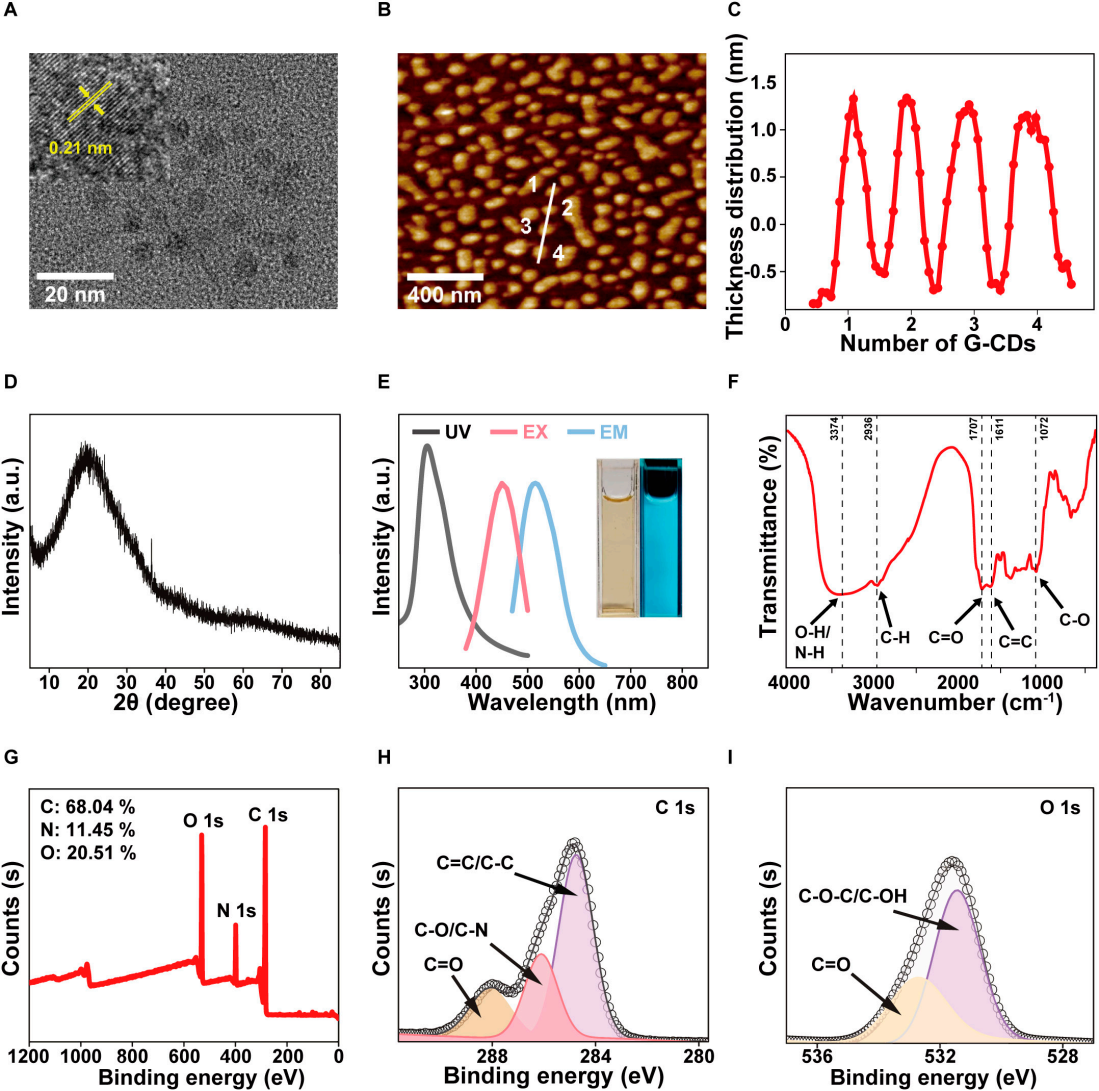
Characteristics of G-CDs. (A) TEM (inset: high-resolution TEM) image of G-CDs. (B and C) AFM image and thickness distribution of G-CDs. (D) XRD pattern of G-CDs. (E) UV absorption spectra (black line), fluorescence excitation (red line), and emission (blue line) spectra of G-CDs. Inset: Images of G-CDs taken under daylight (left) and 365-nm UV lamp (right). (F) FTIR spectra of G-CDs. (G) Full-scan XPS spectrum of G-CDs. (H and I) High-resolution C 1s and O 1s XPS spectra of G-CDs.

To avoid hazardous solvents during the synthesis process, G-CDs with anti-inflammatory and antioxidant activity were prepared from *Glycyrrhiza* using a hydrothermal method (Fig. S1). When these G-CDs were examined via TEM, they were found to be homogeneous in terms of their size and to be highly dispersible (Fig. 2A), with an average particle size of 4.67 ± 1.2 nm (Fig. S2A). High-resolution TEM images (Fig. 2A, inset) revealed the crystallinity of these G-CDs, which exhibited clear 0.21-nm lattice fringes that were consistent with the (100) crystal plane of carbon [17]. AFM was additionally used to assess the morphology of these G-CDs, revealing that they were uniformly distributed and ranged from 1.5 to 2.0 nm in thickness (Fig. 2B and C). Based on the consistency between this thickness and the average diameter obtained via TEM, these G-CDs appear to be quasi-spherical nanoparticles. The broad XRD peaks were consistent with G-CDs purity, and a diffraction peak observed at ~22° provided further support for the existence of the carbon crystal plane [18], in line with the high-resolution TEM results (Fig. 2D). As shown in Fig. 2E, the optical properties of the prepared G-CDs were excellent, exhibiting an ultraviolet–visible (UV–Vis) absorption peak at 320 nm that was attributable to the π–π* transition of the sp^2^ domains in the carbon structure [19]. When the fluorescence spectra of these G-CDs were examined, the optimal excitation and emission wavelengths were respectively measured at 450 and 515 nm (Fig. S2B). By using an integrating sphere system, the absolute quantum yield of G-CDs was determined as ≈5.6%, indicating the bioimaging potential of G-CDs. Under ambient light, G-CDs formed a transparent yellow-colored solution, whereas they emitted bright blue fluorescence when exposed to a 365-nm UV light source (Fig. 2E, inset). G-CDs fluorescence intensity was also consistent across various stability-tested concentrations of NaCl (0 to 1.0 M) (Fig. S2C). The cellular uptake of G-CDs in RAW264.7 cells was detected via fluorescence laser confocal scanning microscopy (LCSM), which revealed the internalization of these G-CDs in RAW264.7 cells within 10 min. The resultant fluorescence intensity was thereafter stable for a minimum of 30 min (Fig. S3).

FTIR spectroscopy and XPS were next used to examine the surface chemistry and functional groups associated with these G-CDs. In FTIR spectra, distinct peaks were evident at 3,374 cm^−1^ (corresponding to the stretching vibrations of -OH or -NH), 2,936 cm^−1^ (C–H stretching), 1,707 cm^−1^ (C=O stretching), 1,611 cm^−1^ (C=C stretching), and 1,072 cm^−1^ (C–O stretching), consistent with functional groups being present on the surfaces of these G-CDs (Fig. 2F). In XPS analyses (Fig. 2G), these nanoparticles were found to consist primarily of carbon (68.04%), oxygen (20.51%), and nitrogen (11.45%). High-resolution XPS spectra revealed the deconvolution of the C1s spectrum into 3 major peaks at binding energies of 284.8, 286.1, and 288.1 eV, respectively corresponding to C=C/C–C, C–O/C–N, and C=O (Fig. 2H). The deconvolution of the N1s spectrum also yielded peaks at 399.8 and 401.6 eV that were respectively associated with pyrrolic nitrogen and graphitic nitrogen (Fig. S4). The O1s spectrum exhibited peaks at 531.4 and 532.7 eV that were respectively associated with C–O–C/C–OH and C=O bonds (Fig. 2I). These findings offer detailed insight into the chemical structures and surface functional groups associated with these G-CDs, confirming the successful preparation of spherical-like G-CDs with many unsaturated surface functional groups, excellent crystallinity, and fluorescence properties.

### Evaluation of the SOD-like nanozyme activity of G-CDs

**Fig. 3.**
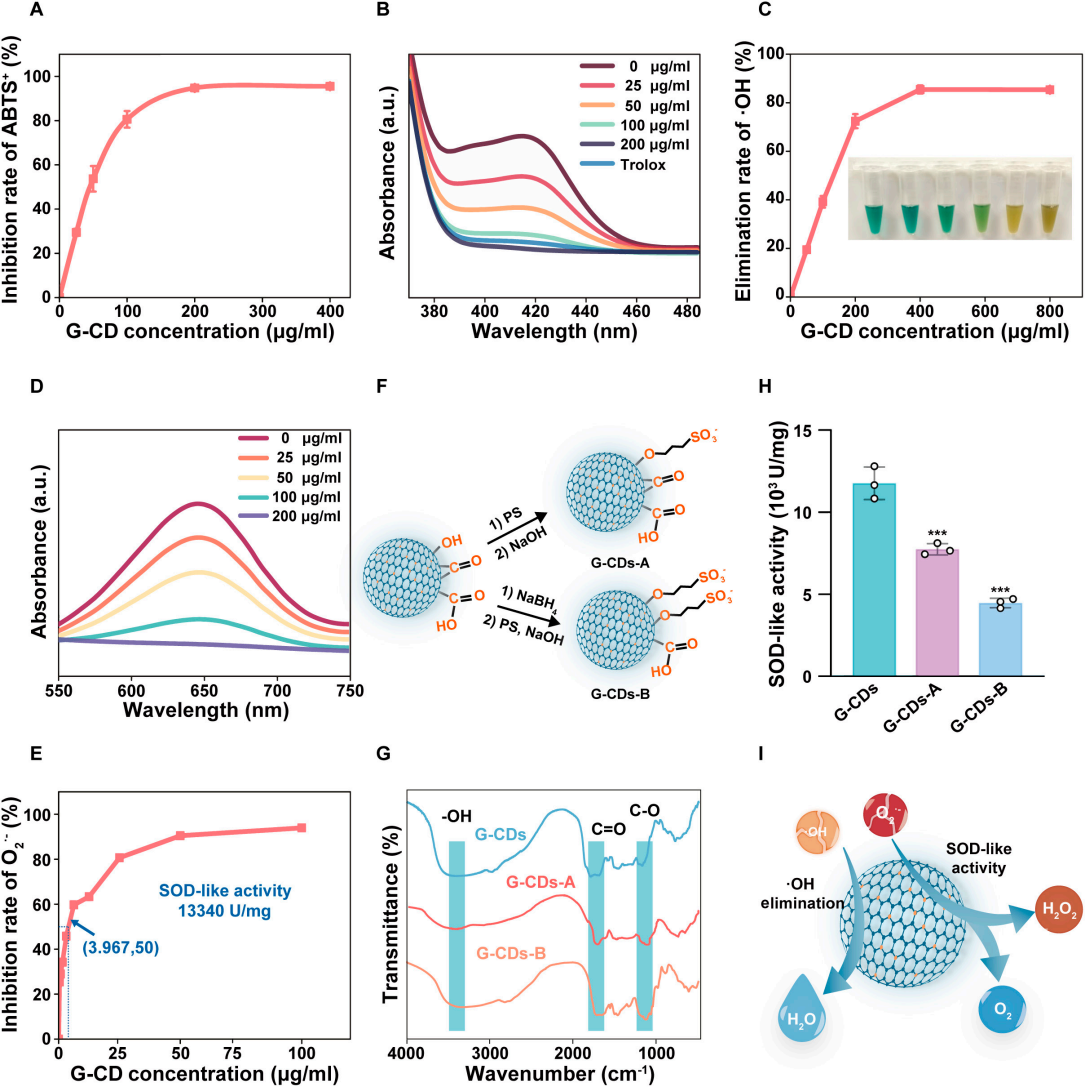
SOD-like activity of G-CDs. (A) The total antioxidant capacity of G-CDs was measured by using the ABTS method. (B) UV–Vis absorption spectra of ABTS^•+^ solutions after mixing with G-CDs at different concentrations. (C) The •OH scavenging capacity of G-CDs was detected based on the Fenton reaction. (D) UV–Vis absorption spectra of TMB solutions after mixing with G-CDs. (E) SOD-like activity of G-CDs. (F) Illustration of G-CDs modification. (G) FTIR spectra of G-CDs, G-CDs-A, and G-CDs-B. (H) SOD-like activities of G-CDs, G-CDs-A, and G-CDs-B. (I) Schematic diagram of the free radical scavenging activity of the G-CDs nanozyme. ***P < 0.001.

As these G-CDs harbored many unsaturated functional groups on their surfaces, this was suggestive of their potential for robust antioxidant activity. We initially employed an ABTS method to test the total antioxidant capacities of G-CDs. When exposed to oxidants, ABTS can undergo oxidation to produce ABTS^•+^, which has a distinct absorption peak at 414 nm [20]. This ABTS^•+^ formation process can be inhibited when antioxidants are present, and in assays focused on total antioxidant capacity, G-CDs were found to inhibit ABTS^•+^ generation in a dose-dependent manner, with 200 μg/ml of G-CDs presenting with inhibitory activity comparable to that of a 50 μM positive control dose (Fig. 3A and B). The cellular ROS are mainly composed of hydroxyl radicals (•OH), superoxide anion (O_2_^•−^), and hydrogen peroxide (H_2_O_2_), which are usually generated as by-products of aerobic metabolism. We assayed the scavenging of •OH by G-CDs based on the Fenton reaction, with Fe^2+^ present in the reaction catalyzing the generation of large quantities of hydroxyl radicals from H_2_O_2_; after that, the chromogenic substrate TMB can react with these radicals to produce a colored product that can be measured based on its characteristic absorption peak at 652 nm. G-CDs were able to efficiently scavenge hydroxyl radicals, removing more than 70% of these radicals at a concentration of 200 μg/ml (Fig. 3C and D). Based on these findings, G-CDs exhibit highly potent antioxidant activity. Superoxide dismutase (SOD) is a key antioxidant enzyme responsible for superoxide anion neutralization [21]. G-CDs were found to exhibit robust O_2_^•−^ scavenging activity in NBT and WST assays (Fig. S5), inhibiting 50% of the WST-1 and O_2_^•−^ reactions at a G-CDs concentration of 3.75 μg/ml (Fig. 3E). In the WST detection analyses, the SOD-like activity of these G-CDs was measured at 13,340 U/mg such that these naturally derived preparation nanoparticles exhibit high SOD nanozyme activity levels.

The role of surface hydroxyl groups in this observed SOD-like activity was tested by selectively deactivating these functional groups using PS, which reacts with carboxyl and hydroxyl groups to form ester and ether bonds. Under alkaline conditions, the ester bonds undergo hydrolysis, whereas the ether bonds remain intact [22]. Following the hydrolysis of PS-treated G-CDs in 0.5 M NaOH, G-CDs-A samples in which the hydroxyl groups had been passivated were obtained (Fig. 3F). The FTIR spectral analysis presented in Fig. 3G exhibited a decrease in absorption intensity within O-H (3,374 cm^−1^), while the absorption of C–O (1,072 cm^−1^) was increased, suggesting that the hydroxyl and amide have reacted with PS. In addition, the importance of surface carbonyls for the SOD-like activity of G-CDs was assessed by their chemical reduction with NaBH_4_ (Fig. 3F). In the FTIR spectrum of G-CDs-B (Fig. 3G), the absorption of C–O increased, followed by a decrease in the absorption of C=O (1,707 cm^−1^), indicating that the carbonyl groups were reduced to hydroxyl groups. In the WST assay, distinctly weaker SOD-like activity was observed for G-CDs-A and G-CDs-B, emphasizing the key roles that carbonyl and hydroxyl groups play as mediators of this SOD-like nanozyme activity (Fig. 3H). Together, these results provide support for the ability of G-CDs to scavenge free radicals through their SOD-like activity mediated by hydroxyl and carbonyl groups on the surface of these nanoparticles (Fig. 3I).

### G-CDs eliminate ROS and preserve mitochondrial function

**Fig. 4.**
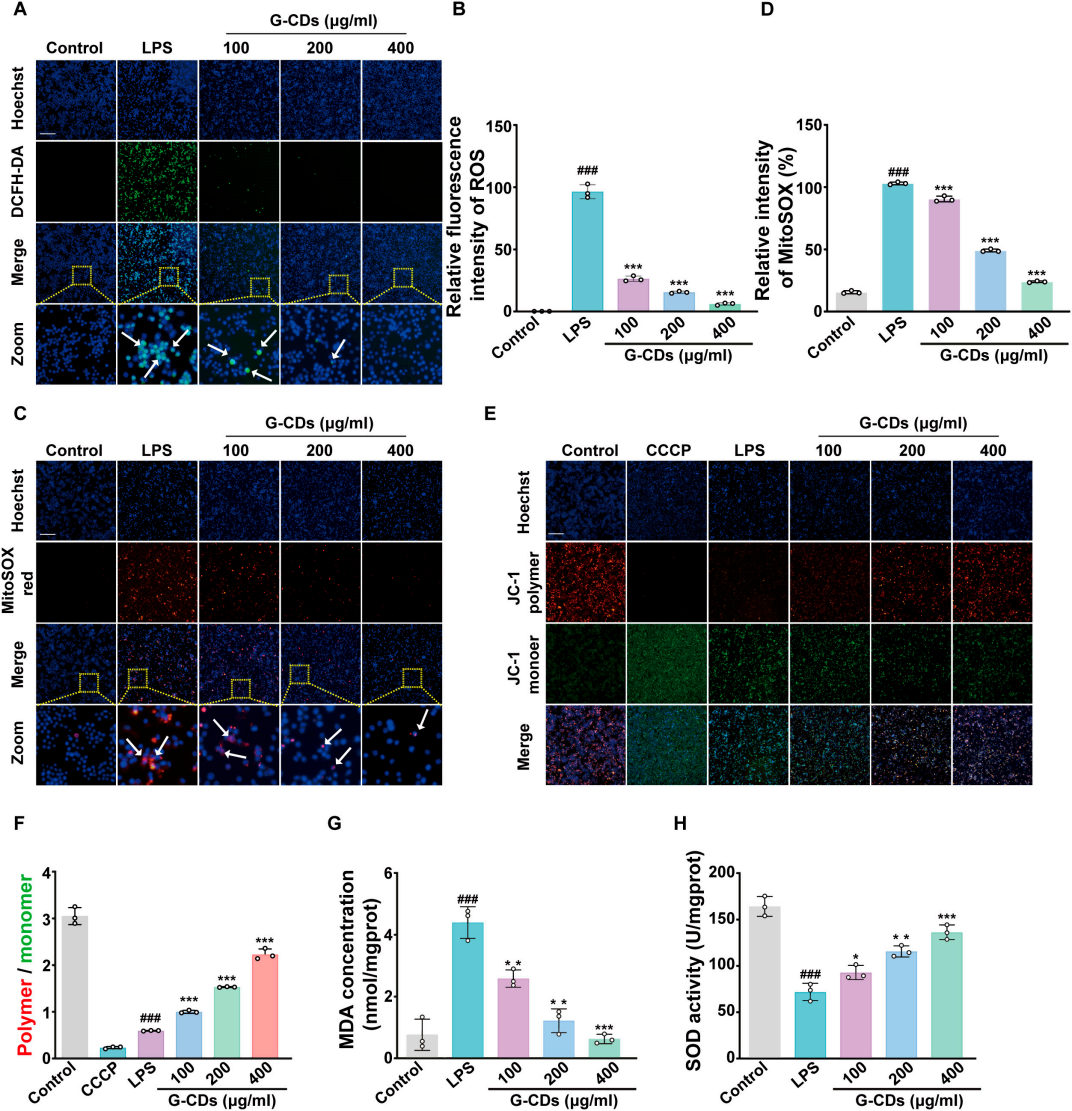
G-CDs eliminate ROS and protect mitochondrial function. (A and B) Detection of ROS using DCFH-DA and Hoechst staining, with quantification of representative fluorescence images; the white arrow represents cellular ROS (scale bar, 200 μm). (C and D) Detection of mitochondrial superoxide using MitoSOX red and Hoechst staining, with quantification of representative fluorescence images; the white arrow represents mitochondrial superoxide (scale bar, 200 μm). (E and F) MMPs in RAW264.7 cells were examined by JC-1 and Hoechst staining, with quantification of representative fluorescence images (scale bar, 200 μm). (G and H) Assessment of cellular MDA and SOD levels. Data are expressed as mean ± SD. *^###^P* < 0.001 versus control group; *P < 0.05, **P < 0.01, ***P < 0.001 versus LPS group; *n* = 3.

The infection-triggered excessive ROS and uncontrolled inflammatory response cause cell damage are critical to the progression of SILI [23]. Given the robust free radical scavenging capacity of these G-CDs in the above experiments, the therapeutic potential was next explored using LPS-stimulated RAW264.7 cells by the DCFH-DA probe. Intense green fluorescence was evident in LPS-treated RAW264.7 cells, consistent with high levels of ROS production, whereas G-CDs dramatically decreased such ROS production as evidenced by a drop in green fluorescence intensity (Fig. 4A and B).

Mitochondria are the organelles responsible for producing most free radicals in each cell, and they can become dysfunctional if ROS accumulate to deleterious levels [24]. A MitoSOX kit was thus used to examine how G-CDs affect mitochondrial function. A substantial increase in superoxide production was noted in LPS-treated cells, whereas the corresponding red fluorescent signal became less intense in G-CDs-treated cells, consistent with the attenuation of mitochondrial ROS levels (Fig. 4C and D). JC-1 staining was also used to quantify MMP, demonstrating the ability of G-CDs to counteract the LPS-induced drop in MMP as determined based on the ratio of red to green fluorescence, highlighting the ability of these nanoparticles to protect against mitochondrial injury caused by LPS (Fig. 4E and F). The lipid peroxidation by-product malondialdehyde (MDA) can be used as an indirect indicator of oxidative injury in cells [25]. The combined assay of MDA and cellular SOD activity can provide a more comprehensive picture of the degree of oxidative stress in specific cells. The cells treated with G-CDs showed reduced MDA accumulation and enhanced SOD activity, consistent with the ability of the nanoparticles to protect against oxidative injury (Fig. 4G and H). In summary, the present results demonstrate the potent antioxidant properties of G-CDs given their ability to limit the production of ROS and to preserve the functionality of mitochondria.

### G-CDs inhibit NF-κB signaling to limit inflammation

**Fig. 5.**
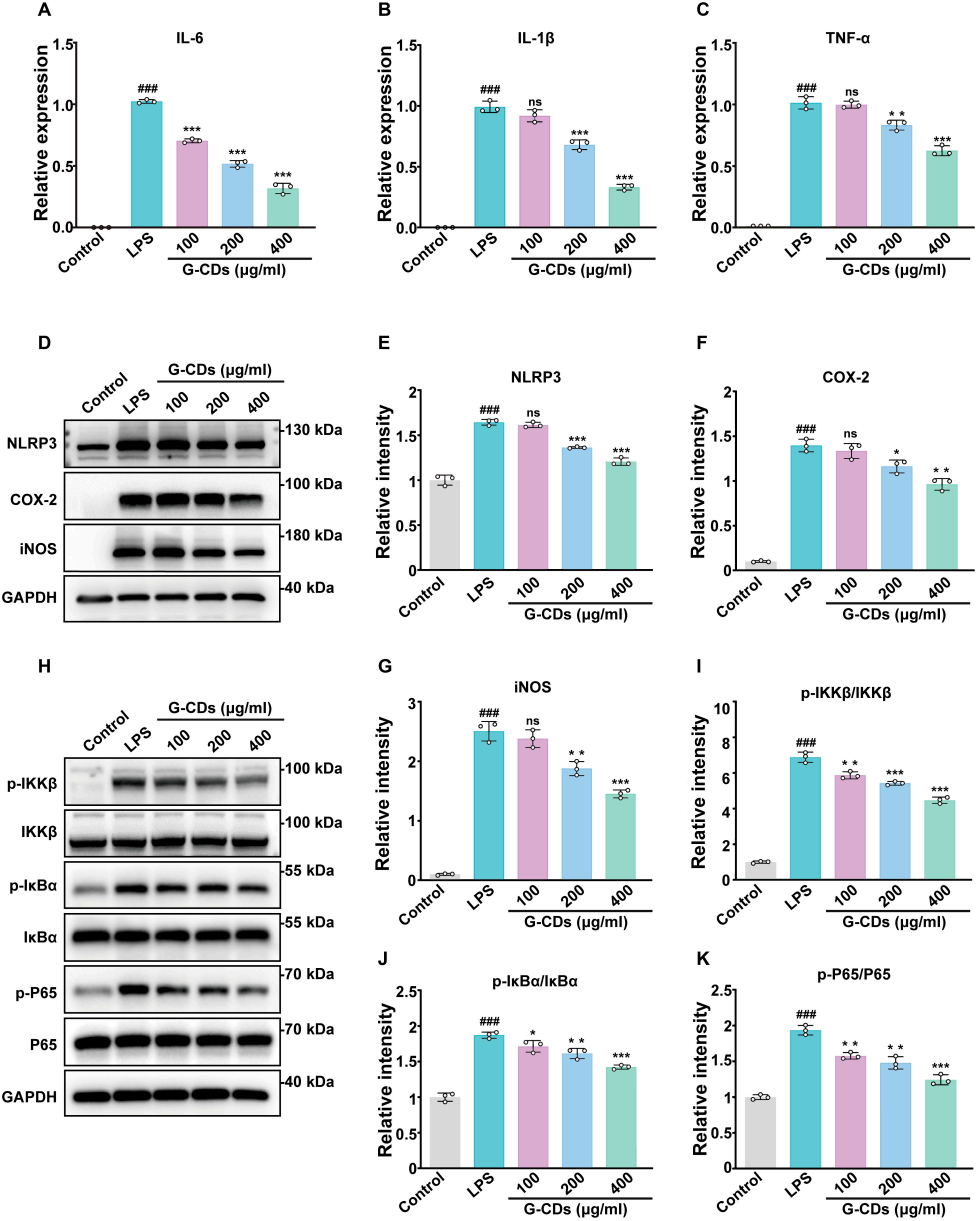
G-CDs exert anti-inflammatory effects by inhibiting the NF-κB pathway. (A to C) RAW264.7 cells were treated with LPS (1 μg/ml) in the absence or presence of G-CDs for 6 h. The mRNA expression levels of IL-6, IL-1β, and TNF-α were measured by qPCR. (D to G) Representative images and quantification of Western blotting analysis showing the levels of NLRP3, COX-2, and iNOS in the presence or absence of G-CDs treatment. (H to K) Representative images and quantification of Western blotting analysis showing the levels of phosphorylated IKKβ (p-IKKβ), phosphorylated IκBα (p-IκBα), and phosphorylated P65 (p-P65) in the presence or absence of G-CDs treatment. Quantitative analysis from 3 independent experiments. Data are expressed as means ± SD. *^###^P* < 0.001 versus control group; *P < 0.05, **P < 0.01, ***P < 0.001 versus LPS group; ns, not significant; *n* = 3.

The data shown above highlight the ability of G-CDs to readily clear ROS from LPS-treated cells, and overly high levels of oxidative stress are closely linked to inflammatory responses mediated by macrophages. When the anti-inflammatory effects of these G-CDs were further explored at the cellular level, treatment for 24 h with G-CDs was sufficient to suppress LPS-induced NO release in a dose-dependent fashion (Fig. S6A). In quantitative polymerase chain reaction (qPCR) analyses, G-CDs further inhibited M1 polarization-associated gene up-regulation in these macrophages, including the levels of the transcripts encoding interleukin-6 (IL-6), IL-1β, and tumor necrosis factor-α (TNF-α) (Fig. 5A to C). In contrast, these G-CDs had no impact on the expression of the M2 marker genes Arg-1 and CD206 (Fig. S6B and C), indicating that they may exert their effects primarily through their ability to alter the M1 polarization of macrophages. Furthermore, G-CDs also suppressed the up-regulation of inflammatory proteins such as COX-2, iNOS, and NLRP3 (Fig. 5D to G). The NF-κB signaling pathway is central to the coordination of inflammatory cellular responses triggered by external stimuli. IKKβ is phosphorylated in response to LPS, leading to IκBα phosphorylation and nuclear p65 translocation, culminating in the up-regulation of pro-inflammatory mediators [26]. Western immunoblotting demonstrated the ability of G-CDs to inhibit IKKβ phosphorylation dose-dependently (Fig. 5H and I). G-CDs also decreased IκBα and NF-κB p65 phosphorylation in this assay system (Fig. 5H, J, and K), highlighting their ability to reliably suppress LPS-induced NF-κB pathway activation.

In conclusion, G-CDs appear to present with excellent anti-inflammatory activities that entail the inhibition of M1 macrophage polarization and the suppression of NF-κB pathway activation. These nanoparticles are therefore promising candidate drugs for the treatment of inflammatory diseases such as SILI.

### G-CDs activate the Keap1/Nrf2 pathway to exert their antioxidant effects

**Fig. 6.**
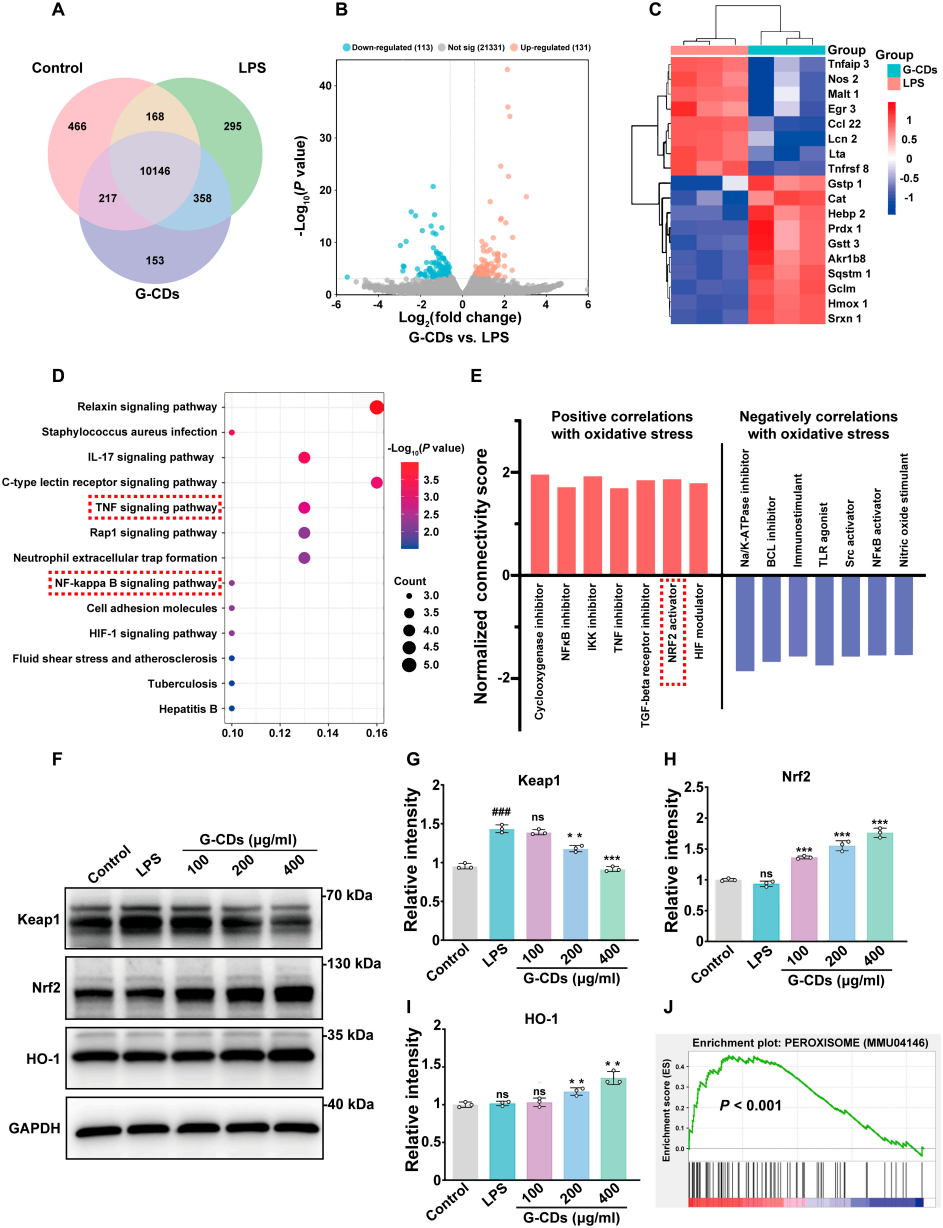
G-CDs exert antioxidant effects by activating the Keap1/Nrf2 pathway. (A) Venn diagram of common and specific genes in the control, LPS, and G-CDs groups. (B) Volcano plot of DEGs in the G-CDs group compared to the LPS group, with the left side representing down-regulated DEGs and the right side representing up-regulated DEGs. (C) Heatmap of inflammation- and oxidative stress-related genes. (D) Compared to the LPS group, the down-regulated pathways in the G-CDs group are mostly related to inflammatory responses. (E) Connectivity map instances (or datasets) and functional annotations of small molecules with the most significant positive and negative correlations with G-CDs activity. The connectivity score shown on the *y* axis is a measure of correlation strength. (F to I) Representative images and quantification of Western blotting analysis showing the levels of Keap1, Nrf2, and HO-1 in RAW264.7 cells in the presence or absence of G-CDs treatment. (J) GSEA plots of peroxisome. Quantitative analysis from 3 independent experiments. Data are expressed as means ± SD. *^###^P* < 0.001 versus control group; **P < 0.01, ***P < 0.001 versus LPS group; *n* = 3.

To better clarify the mechanistic basis for the antioxidant activity of these G-CDs, RNA-seq analyses of RAW264.7 cells treated for 6 h with or without G-CDs were next performed. Relative to control cells, LPS-treated cells exhibited markedly altered gene expression patterns (Fig. 6A). When comparing the LPS + G-CDs and LPS treatment groups, 244 differentially expressed genes (DEGs) were identified (131 up-regulated, 113 down-regulated; fold change > 1.5, *P* < 0.01) (Fig. 6B). The results were arranged in a clustering heatmap revealing many genes associated with antioxidant stress and inflammatory responses among these DEGs (Fig. 6C). Gene Ontology (GO) enrichment of the DEGs up-regulated following G-CDs treatment revealed that they were closely associated with free radical clearance and MMP regulation, whereas the down-regulated DEGs were associated with oxidative stress-related biological processes, supporting the ability of G-CDs to protect against oxidative injury (Fig. S7). Kyoto Encyclopedia of Genes and Genomes (KEGG) enrichment analyses revealed the ability of G-CDs to regulate inflammation-related signaling, particularly with respect to the NF-κB and TNF signaling pathways (Fig. 6D).

The cMAP database has been developed to facilitate the analysis of small-molecule drug-related changes in gene expression. This database can enable rapid comparisons of gene expression profiles to identify drugs closely associated with diseases while also enabling researchers to draw inferences regarding the mechanisms through which a given drug may function [27]. In Fig. 6E, 7 small-molecule functions that were the most strongly positively correlated with oxidative stress are shown along the left side. Of these functions, functional annotation results revealed a particularly close relationship between Nrf2 activators and oxidative stress, supporting the potential status of Nrf2 as a target through which G-CDs exert their therapeutic effects. Accordingly, target proteins in the Nrf2 pathway were next examined, revealing the ability of G-CDs to reduce the levels of the upstream protein Keap1 while significantly enhancing Nrf2 expression and the expression of its downstream target HO-1 (Fig. 6F to I). ‌Gene Set Enrichment Analysis (GSEA) analyses further revealed the enrichment of many peroxisome-related genes in the G-CDs treatment group as compared to the LPS group, attesting to the antioxidant effects of these G-CDs (Fig. 6J). These results support a model wherein G-CDs can control inflammation and exert their antioxidant effects through Keap1/Nrf2 pathway activation.

### In vivo validation of the therapeutic efficacy of G-CDs

**Fig. 7.**
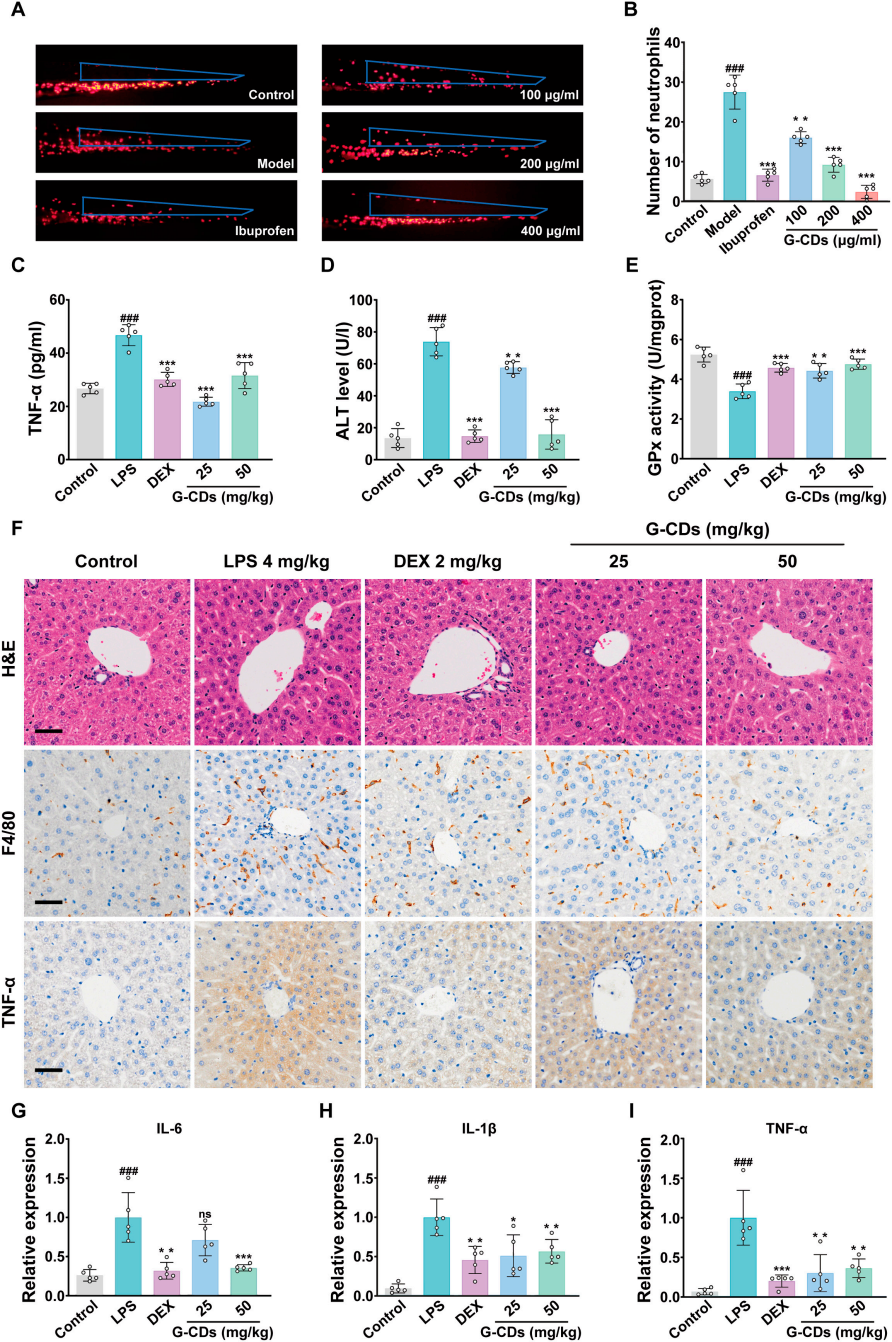
Anti-inflammatory activity of G-CDs in vivo. (A and B) Phenotypes and quantitative analysis of neutrophil distribution and tail neutrophil spread to lateral line in zebrafish. The blue box indicates the neutrophil spreading area. (C) TNF-α levels in mouse sera, measured by enzyme-linked immunosorbent assay (ELISA) kit. (D) ALT levels in mouse sera. (E) Activity of GPx in mouse liver tissues. (F) Representative images of H&E staining and F4/80 and TNF-α immunohistochemistry of mouse liver tissue. Scale bar, 100 μm. (G to I) mRNA expression levels of IL-6, IL-1β, and TNF-α in mouse liver tissue, measured by qPCR. Data are expressed as means ± SD. *^###^P* < 0.001 versus control group; *P < 0.05, **P < 0.01, ***P < 0.001 versus LPS group; *n* = 5.

The above in vitro data offer clear support for the antioxidant and anti-inflammatory properties of the prepared G-CDs. To confirm these findings in vivo, a zebrafish model of acute inflammation was first established (Fig. S8). CuSO₄ exposure caused substantial acute injury and inflammation in these fish characterized by red fluorescent neutrophil migration from the dorsal and abdominal aorta to the lateral line neural thalamus [28]. While a considerable increase in such neutrophil migration was apparent in the CuSO₄ model group, it was readily suppressed by ibuprofen or G-CDs, with a 400 μg/ml dose of G-CDs exhibiting anti-inflammatory activity levels analogous to those observed for 20 μM ibuprofen (Fig. 7A and B), underscoring the potent in vivo antioxidant activity of G-CDs.

Considering this promising activity in zebrafish, a murine model of SILI was established to evaluate the therapeutic utility of G-CDs. As LPS can induce inflammatory cytokine secretion from hepatic macrophages, triggering the necrotic or apoptotic death of these cells and associated liver injury [29], SILI modeling was achieved via the intraperitoneal injection of LPS. Mice in appropriate groups were pretreated for 3 d with G-CDs before experimental initiation and received a dose of these G-CDs intraperitoneally for 1 h before LPS was injected, with dexamethasone serving as a positive control (Fig. S9). G-CD treatment was associated with substantially reduced serum levels of the inflammatory factor TNF-α (Fig. 7C). Serum ALT levels, which are indicative of hepatic damage, were also significantly lower in the G-CDs treatment group relative to the LPS group (Fig. 7D), together with observed significant tissue up-regulation of the antioxidant enzyme glutathione peroxidase (GPx) (Fig. 7E). Furthermore, the mice in the model group exhibited dull, unkempt fur and reduced activity, while those in the LPS + G-CDs group showed gradual improvement in appearance and vitality with increasing doses of G-CDs (Fig. S10). As demonstrated in Fig. 7F, histopathological staining of the liver tissue was also performed, revealing a marked decrease in inflammatory cell infiltration in the G-CDs group relative to the LPS model group. Immunohistochemical staining for F4/80 and TNF-α also demonstrated significant reductions in immune cell infiltration in the liver of G-CDs-treated animals (Fig. 7F). Consistently, the hepatic expression of inflammatory genes (IL-6, TNF-α, and IL-1β) was significantly reduced by the administration of G-CDs relative to the LPS model group (Fig. 7G and H). These data indicate that relative to model animals, G-CD treatment effectively alleviated inflammation and hepatic damage, with high-dose G-CDs offering efficacy levels analogous to those of 2 mg/kg dexamethasone. These G-CDs can thus protect against SILI by suppressing inflammatory cytokine production while simultaneously augmenting antioxidant defenses.

### Assessment of G-CDs biosafety

**Fig. 8.**
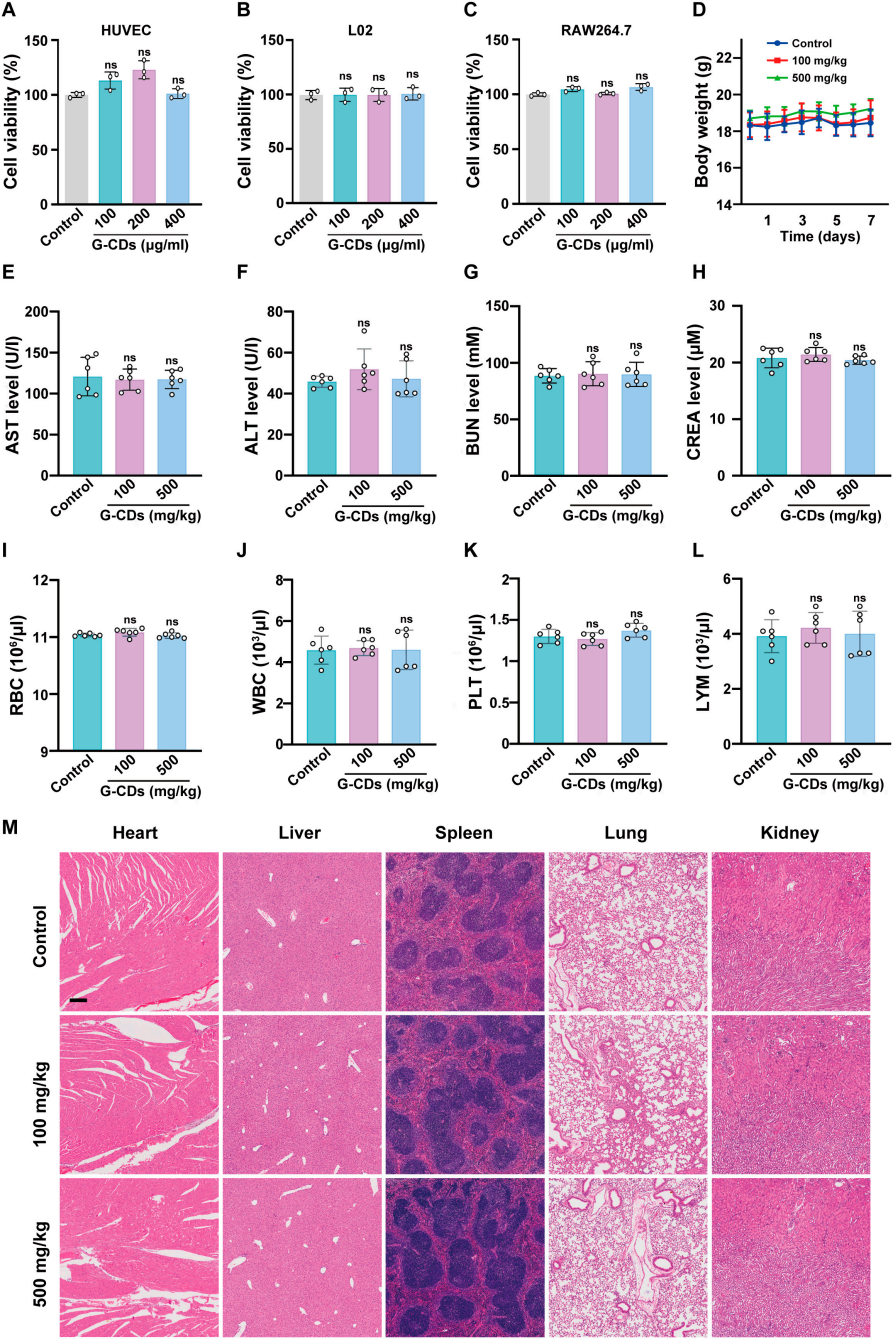
Biosafety verification of G-CDs. (A to C) CCK-8 analysis was performed with various concentrations of G-CDs in RAW264.7, HUVEC, and L02 cells (*n* = 3). (D) Continuous monitoring of changes in mouse body weights. (E to H) Blood biochemical indices (AST, ALT, BUN, and CREA) in mice following administration of G-CDs by gavage for 7 d. (I to L) Routine blood indicators [red blood cells (RBC), white blood cells (WBC), platelets (PLT), and lymphocytes (LYM)] in mice following administration of G-CDs by gavage for 7 d. (M) Representative images of the histologic morphology of different tissues (heart, liver, spleen, lungs, and kidneys) were evaluated by H&E staining. Scale bar, 100 μm. The data are expressed as mean ± SD. *n* = 6.

To determine the feasibility of applying G-CDs as therapeutic anti-inflammatory agents in a biomedical context, their biocompatibility was next tested. These G-CDs did not cause any apparent cytotoxicity when used to treat human umbilical cord endothelial cells (HUVECs), L02 cells, and RAW264.7 cells at concentrations ranging from 0 to 400 μg/ml, as determined in a CCK-8 assay (Fig. 8A to C).

The acute toxicity of G-CDs was also assessed by administering a single dose of these nanoparticles (100 and 500 mg/kg) to BALB/c mice and then continuously monitoring these animals for 7 d. No significant changes in body weight were noted when comparing the G-CDs treatment group and the normal control group over this period (Fig. 8D). Serum ALT, AST, CREA, and BUN levels were also comparable in the G-CDs treatment and control groups, consistent with the absence of any hepatotoxicity or nephrotoxicity (Fig. 8E to H). Peripheral blood analyses also revealed that the routine blood parameters in these mice were not significantly altered by G-CDs administration, indicating that these nanoparticles do not affect hematological function (Fig. 8I to L). When H&E staining was used to assess the histopathology of major organs including the heart, lungs, kidneys, liver, and spleen, no abnormalities were apparent in any treatment group (Fig. 8M), indicating that G-CDs cause no significant organ damage or toxic effects (Fig. S11).

These results highlight the excellent biosafety of G-CDs, as they had no apparent toxic effects at any tested dose level. They thus hold promise as a safe and efficacious biotherapeutic agent for the management of SILI and other inflammatory diseases.

## Discussion

Natural plants rich in multiple active ingredients as the ideal green carbon sources for CDs synthesis offer cost-effectiveness and biosafety superior to that of alternative carbon sources [30]. While plant-derived CDs have been shown to effectively treat a wide range of inflammatory diseases [31], the therapeutic potential of plant-derived CDs in treating sepsis remains largely underexplored. In this study, G-CDs were synthesized via hydrothermal treatment for the first time, and their ameliorative effects on septic liver injury were systematically evaluated through in vitro enzyme assays, cellular experiments, histological analysis, and animal models. The underlying mechanisms were also investigated. In this study, G-CDs were synthesized via hydrothermal treatment for the first time, and their ameliorative effects on SILI were systematically evaluated through in vitro enzyme assays, cellular experiments, histological analysis, and animal models and the underlying mechanisms were also investigated. The *Glycyrrhiza*-derived CDs exhibit promising potential in biomedical applications. Qu et al. synthesized *Glycyrrhizae* radix et rhizome-derived carbon dots (GRR-CDs) from the rhizome of medicinal *Glycyrrhiza* using high-temperature pyrolysis. These GRR-CDs alleviated gastric ulcers by enhancing systemic antioxidant capacity and protecting the gastric mucosal barrier from ethanol-induced damage [32]. Additionally, GRR-CDs improved menopausal symptoms by up-regulating estradiol levels and preventing uterine atrophy [33]. In this study, hydrothermal synthesis was employed as an alternative to high-temperature pyrolysis, effectively preserving active functional groups such as hydroxyl, carboxyl, and epoxy moieties from the *Glycyrrhiza* extract. These groups condensed with partially carbonized organic molecules during the reaction, forming functionalized surface modifications on G-CDs [31]. In comparison with Gly-CDs with excellent antiviral activity previously derived from glycyrrhizic acid by Liang and colleagues [34], our G-CDs demonstrated considerable discrepancies in their physicochemical properties. Specifically, G-CDs possessed a smaller average particle size and higher N content. Nitrogen atoms, which exhibit high electronegativity, have been shown to regulate surface charge distribution, thereby impacting the catalytic performance of CDs [35]. Nitrogen-doped CDs is an effective Fenton-like catalyst for catalyzing the decomposition of H_2_O_2_ in the Fenton reaction system [36]. However, despite the higher N content, G-CDs did not exhibit catalytic activity for H_2_O_2_ decomposition (Fig. S12A), which may be related to the fact that the doped N elements in G-CDs were mostly embedded in the carbon skeleton and failed to form effective reactive groups on the surface. The embedded nitrogen atoms on the backbone of G-CDs may play a role in the •OH scavenging activity and exhibit different activities from Gly-CDs. To explore this mechanism in depth, we will devote ourselves to investigating the effects of different forms of nitrogen doping on the activity of G-CDs to further optimize their properties.

Nanozymes are nanomaterials that possess intrinsic enzyme-like activity and exhibit the characteristics of high durability, low costs, and scalable for mass production. These properties have made nanozymes increasingly important in various applications across different fields [37]. For instance, Zeng et al. synthesized CDs with GSH [glutathione (reduced form)] oxidase-like activity from chlorogenic acid (the primary component in coffee) and demonstrated their strong antitumor activity by inducing ferroptosis in cancer cells [38]. Similarly, Sun et al. developed composite materials from cerium and gold nanoparticles with multi-enzyme-like activities, which were used effectively in the treatment of acute myeloid leukemia [39]. In this study, we report the development of a plant-derived SOD nanozyme, which exhibits significant SOD-like activity, reaching up to 13,340 U/mg. The major functional groups observed on the surface of G-CDs include hydroxyl, carbonyl, and carboxyl groups, which confer good water solubility and ease of modification to the CDs. To investigate the influence of surface functional groups on the SOD-like activity of G-CDs, we passivated the surface functional groups with PS [40]. Consistent with previous research, our study further revealed that the carbonyl group acts as an active catalytic site, which contributes to the SOD-like activity of G-CDs [41]. The carbonyl group may interact with other surface functional groups, leading to stereoelectronic effects that enhance the ability of G-CDs to attract and scavenge free radicals [42]. This suggests that the introduction of groups capable of producing stereoelectronic effects on CDs surfaces may greatly enhance their SOD-like activities. In the future, it is worth exploring the influence of stereoelectronic effects on the SOD-like activity of CDs to gain a deeper understanding of the structure–activity relationship of these nanozymes.

The cellular ROS are mainly composed of O_2_^•−^, H_2_O_2_, and •OH, which are known to induce cellular damage when present in excessive amounts. Under normal conditions, the Nrf2 transcription factor is tightly bound to the Keap1 protein, which is located in the cytoplasm and remains inactive [43]. Upon oxidative stress, excessive ROS induces Nrf2 to dissociate from Keap1, resulting in the translocation of Nrf2 into the nucleus to activate the transcription of downstream antioxidant genes [44]. Herein, we observed elevated protein expression of Keap1 and decreased protein expression of Nrf2 in the LPS-induced group, which aligns with previous findings [45]. The maintenance of redox homeostasis critically depends on enzymatic defense systems—particularly SOD, GPx, and catalase (CAT)—that collectively neutralize reactive oxygen species (ROS) generated during aerobic metabolism [46]. Dysregulation of this delicate balance triggers oxidative cascades culminating in cellular apoptosis [47]. Our investigations revealed that G-CDs execute a hierarchical antioxidant strategy: (a) direct superoxide anion (O_2_^•−^) scavenging via SOD-mimetic activity and (b) indirect hydrogen peroxide (H_2_O_2_) regulation through cellular antioxidant system potentiation (Fig. S12B). Although G-CDs exhibited negligible intrinsic CAT-like activity in cell-free systems, they demonstrated remarkable cytoprotective effects in H_2_O_2_-challenged RAW264.7 macrophages (Fig. S13). This tripartite cellular defense mechanism suggests that G-CDs activate endogenous antioxidant responses rather than directly decomposing H_2_O_2_. The coordinated up-regulation of CAT transcription and GPx enzymatic activity implies involvement of the Nrf2–Keap1–ARE signaling axis, a master regulator of antioxidant gene expression [48]. By enhancing cellular self-defense capacity, G-CDs establish a sustainable redox homeostasis without requiring continuous catalytic intervention—a distinct advantage over conventional nanozymes. Nevertheless, the weak fluorescence intensity of G-CDs in cells and animals poses a major challenge in the study of their cell imaging and distribution in vivo (Fig. S14). In the subsequent study, the fluorescence intensity of G-CDs will be enhanced by the attachment of fluorescent probes. This will facilitate further exploration of the subcellular localization and organ targeting of G-CDs, and will enable a more comprehensive understanding of the mechanism of action of G-CDs.

In conclusion, *Glycyrrhiza* was effectively processed through a hydrothermal method to yield G-CDs, which exhibited notable SOD-like activity, antioxidant properties, and free radical scavenging capabilities in this study. The hydroxyl and carbonyl groups present on the surfaces of these G-CDs were identified as the factors most closely associated with the SOD-like activity of these nanoparticles. In vitro, G-CDs demonstrated the capacity to stabilize MMP while suppressing the up-regulation of inflammatory factors and exhibiting a superior capacity for ROS scavenging. At the molecular level, G-CDs were found to exert their antioxidant and anti-inflammatory effects through the activation of Keap1/Nrf2 signaling and the inhibition of NF-κB signaling, thus protecting cells against oxidative injury and inflammation. G-CDs demonstrated exceptional biocompatibility and noteworthy anti-inflammatory properties in zebrafish models of acute inflammation and murine models of SILI. In conclusion, the present results provide strong evidence in support of the antioxidant and anti-inflammatory properties of G-CDs. These results offer a promising avenue for the further development of safe and efficacious nanomedicines with antioxidant properties suitable for clinical use.

## Data Availability

The data used and/or analyzed during the current study are available from the corresponding author upon reasonable request.
