# Supplementary material for: Targeting Reactive Oxygen Species and Inflammation in Sepsis-Induced Liver Injury with Naturally Derived Superoxide Dismutase-Mimicking Carbon Dots
Source: Biomater Res. 2025 Sep 5;29:0249. doi: 10.34133/bmr.0249 (PMC12411697; doi:10.34133/bmr.0249)
Supplement: Supplementary 1 — Supplementary Methods Figs. S1 to S14 Table S1 [file bmr.0249.f1.docx]

**Supplemental Information**

**Targeting ROS and Inflammation in Sepsis-Induced Liver Injury with Naturally Derived Superoxide Dismutase-Mimicking Carbon Dots**

**Chonglei Zhong^a^**^#^**, Nannan Song^a^**^#^**, Ping Huang^a^, Liwen Han^a^,** **Jiguo Zhang^a^*, Zhiyuan Lu^a^*, Lei Wang^a^***

^a^School of Pharmaceutical Sciences & Institute of Materia Medica, State Key Laboratory of Advanced Drug Delivery and Release Systems, Medical Science and Technology Innovation Center, Shandong First Medical University & Shandong Academy of Medical Sciences, Jinan, 250117, China.

^#^These authors contributed equally to this work.

***Corresponding authors.**

**Contents**

**1. Supplementary Methods**

**2.** **Supplementary Figures**

**3. Supplementary Table**

**Supplementary Methods**

**Cell Culture**

The RAW 264.7, NCM460, and HUVEC cells were purchased from the Shanghai Institute for Biological Sciences and cultured in a recommended medium by ATCC which was supplied with 10% fetal bovine serum (APExBIO Technology LLC, Houston, Texas, USA) and 1% Penicillin-Streptomycin (Beyotime, Shanghai, China) at 37°C in a 5% CO_2_ culture incubator.

**G-CDs uptake assays**

RAW264.7 cells were added to confocal dishes (5.0 × 10⁴/well) and incubated in 1 mL of phenol-free DMEM with 10% FBS and 400 μg/mL G-CDs for 0, 5 min, 10 min, 15 min, 30 min, 60 min, 3 h, 6 h, and 12 h. Unbound G-CDs were then removed by washing two times using PBS, after which an LSM980 confocal microscope (Carl Zeiss AG, Germany) was used to visualize G-CDs uptake at 350 nm.

**CCK-8 assay**

After plating cells overnight in 48-well plates (1 × 10^5^/well), they were treated for 24 h with G-CDs, and a CCK-8 assay (TargetMol, China) was then used to assess viability.

**Nitric oxide (NO) production assays**

An NO assay kit. (Nanjing Jiancheng Bioengineering Institute, China) was used to quantify NO levels in cell supernatants. Briefly, after treating RAW264.7 cells for 24 h with LPS (1 μg/mL) with or without G-CDs (100, 200, or 400 μg/mL), culture supernatants were analyzed with the kit as directed by the manufacturer.

**qPCR**

The RNA collection, cDNA synthesis, and quantitative real-time PCR (qPCR) were performed as previously described. Briefly, qPCR analyses were performed using 400 ng of total RNA per sample, using the HiScript III RT SuperMix for qPCR (Vazyme, Nanjing, China) as directed to prepare cDNA. Then, ChamQ Universal SYBR qPCR Master Mix (Vazyme, Nanjing, China) and the primers listed in Table S1 were used for qPCR assays with the following settings: 95°C for 30 s; 40 cycles of 95°C for 10 s, and 60°C for 30 s. The ΔΔCt method was used to assess relative expression. The primers used in this study are provided in Supplemental Table S1.

**Western immunoblotting**

RAW 264.7 cells were lysed with RIPA buffer on ice, after which the samples were centrifuged, and supernatant protein levels were quantified via BCA assay. After separating 15 µg of protein per sample by SDS-PAGE and transferring these samples onto PVDF membranes, the blots were blocked and incubated overnight with primary antibodies at 4°C. Blots were then washed using TBST, incubated for 1 h at room temperature with secondary antibodies, and developed with an enhanced chemiluminescence solution (Tanon), before visualization with an imaging device (Tanion, Shanghai, China).

**Supplementary Figures**


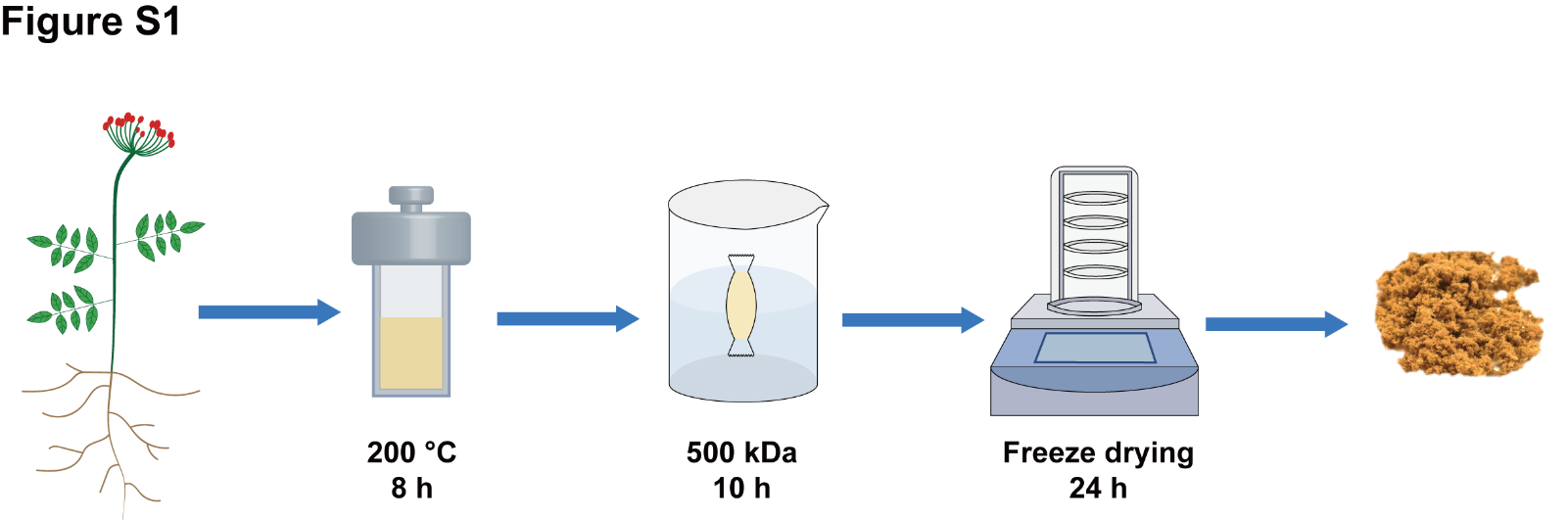


**Figure S1.** Preparation of G-CDs.


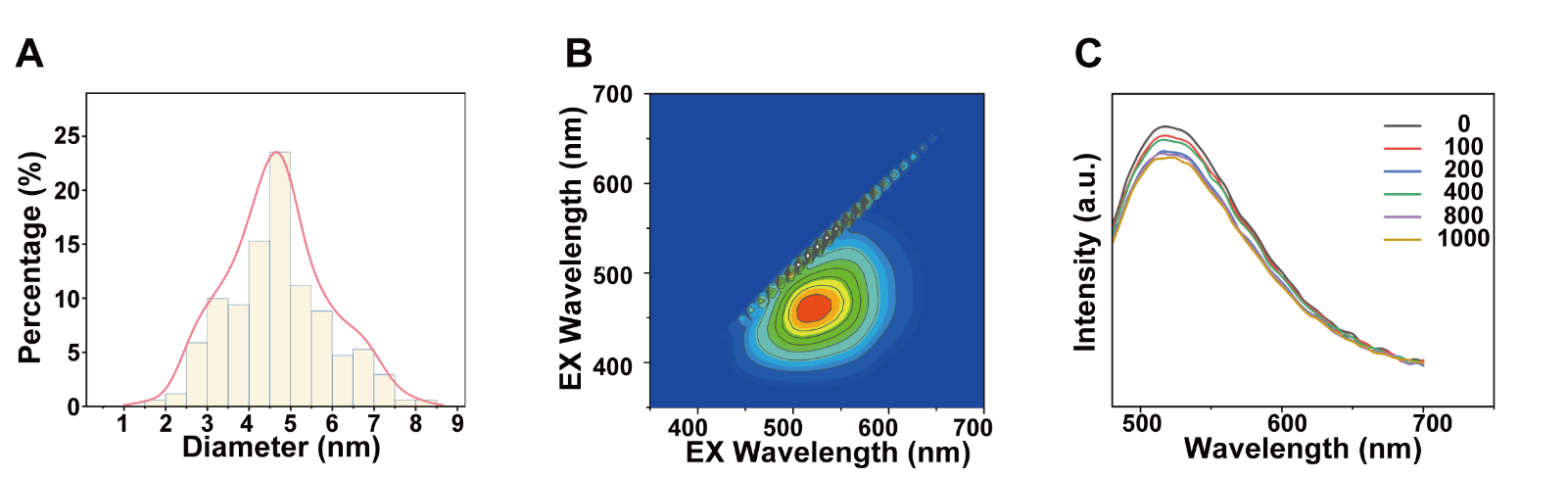


**Figure S2.** **Characteristics of G-CDs.** (A) Particle size distribution. (B) 3D fluorescence spectroscopy. (C) Stability of G-CDs in salt solutions.


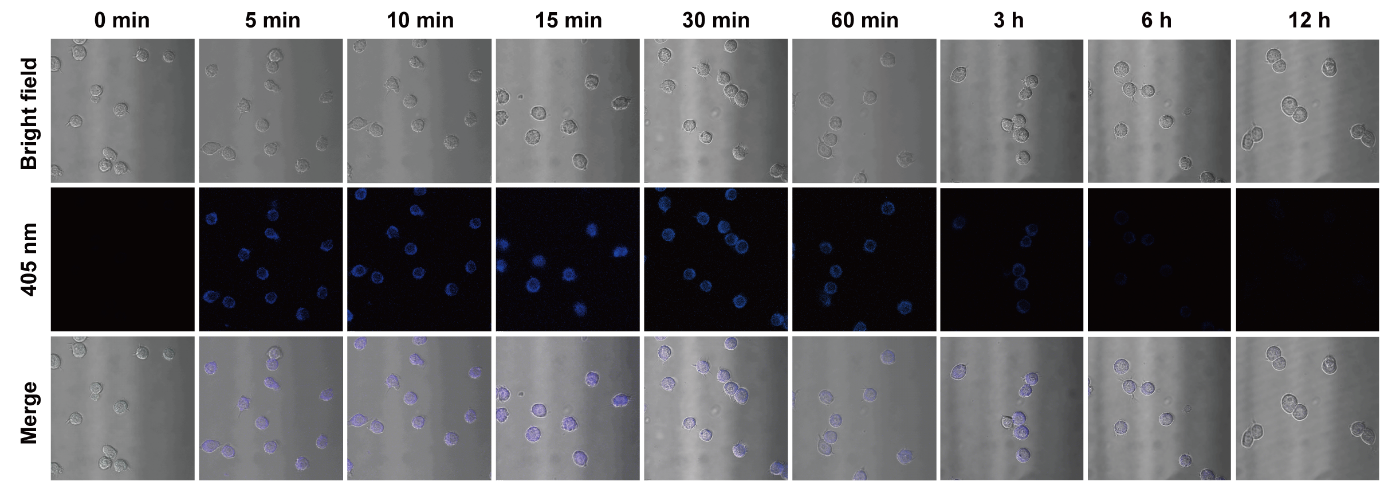


**Figure S3.** The RAW264.7 Cellular Uptake of G-CDs Assay (scale bar = 20 μm).


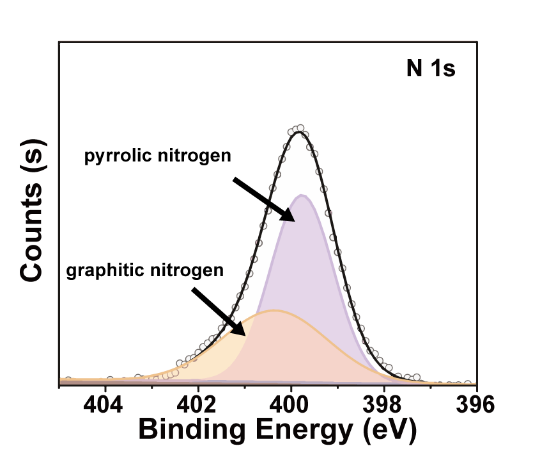


**Figure S4.** High-resolution N 1s XPS spectrum of G-CDs.


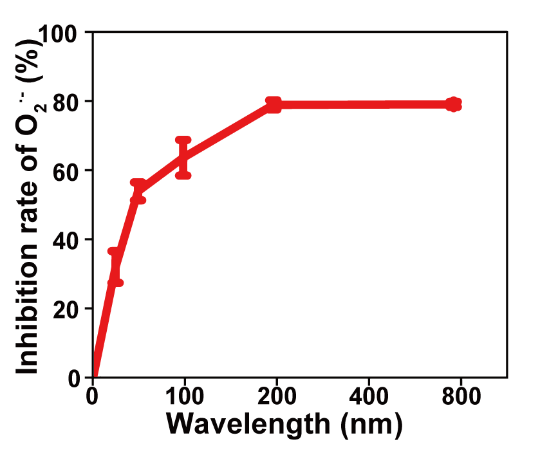


**Figure S5.** NBT method for detecting O_2_^·-^ removal, n=3.


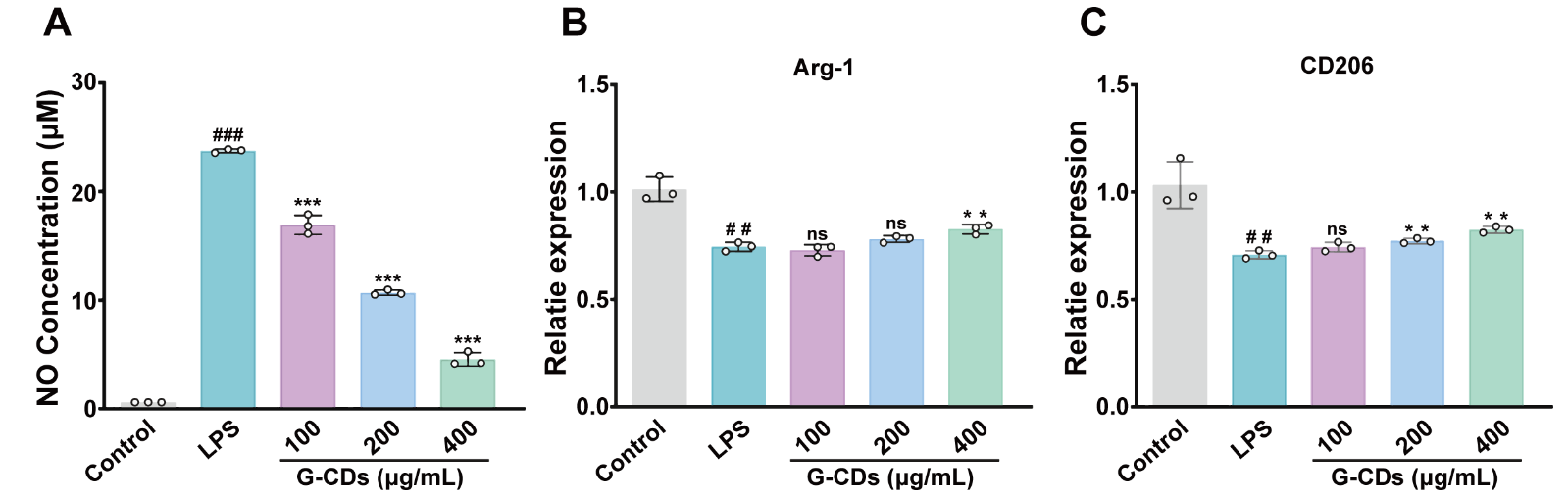


**Figure S6. Effect of G-CDs on NO release and M2 polarization in RAW264.7 cells.** (A) Measurement of NO release. (B, C) The mRNA expression levels of Arg-1 and CD206 were measured by qPCR, *^###^P<0.001*, vs. control group*; *P<0.05, **P<0.01, ***P<0.001,* vs. LPS group, NS means not significant, n=3.


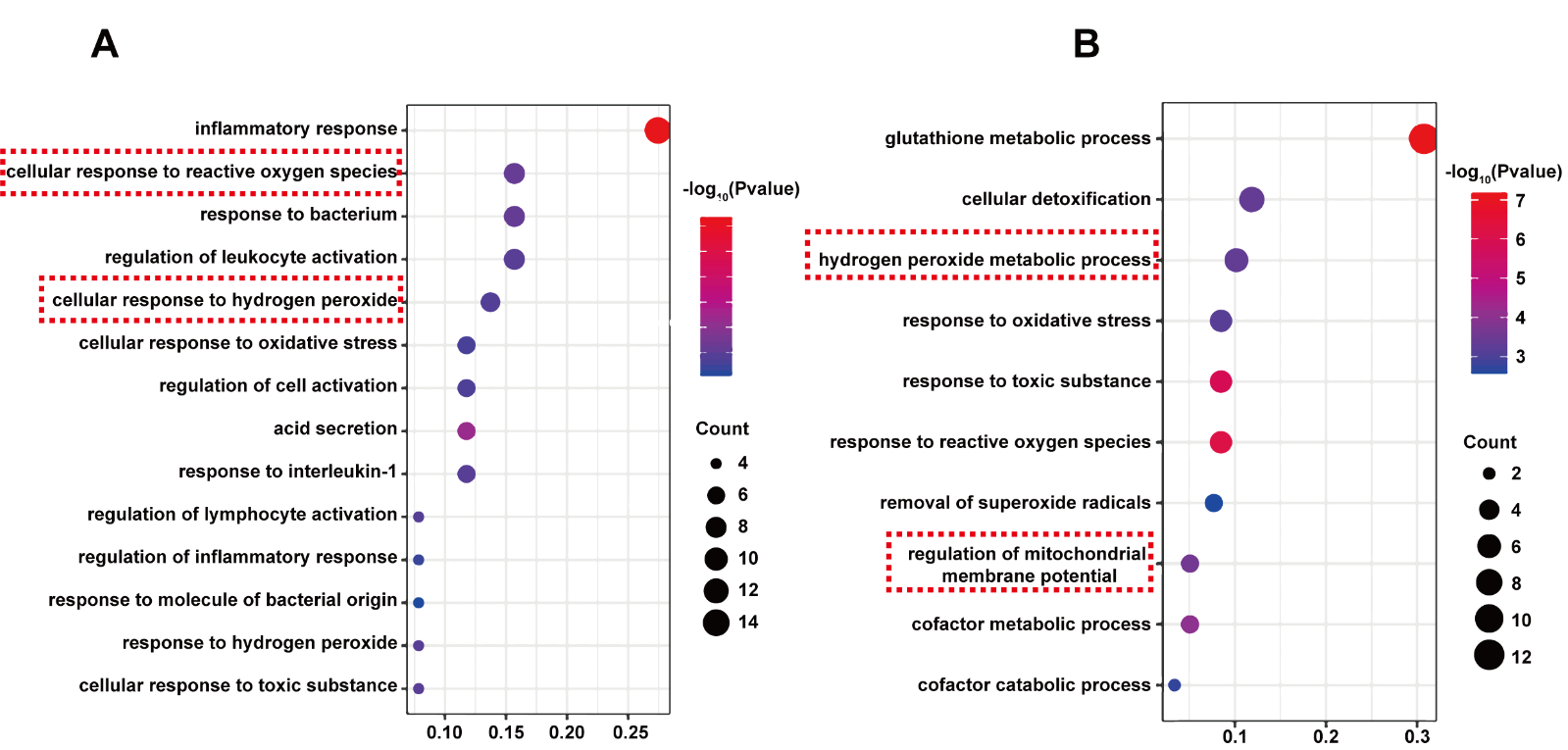


**Figure S7.** Gene Ontology analysis of differentially expressed genes, (A) Down. (B) Up.


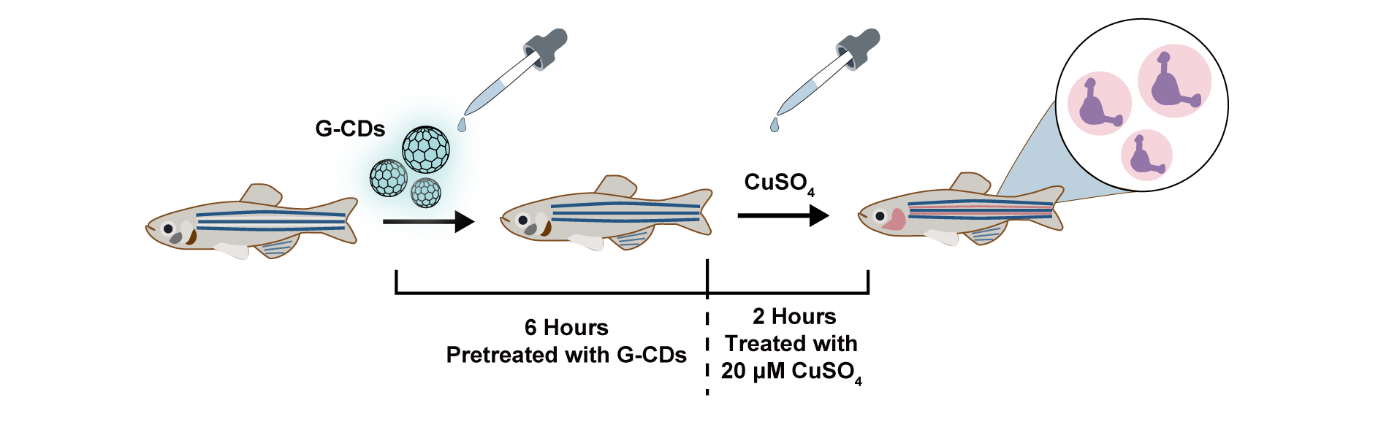


**Figure S8.** Experimental design of the acute inflammation zebrafish model.


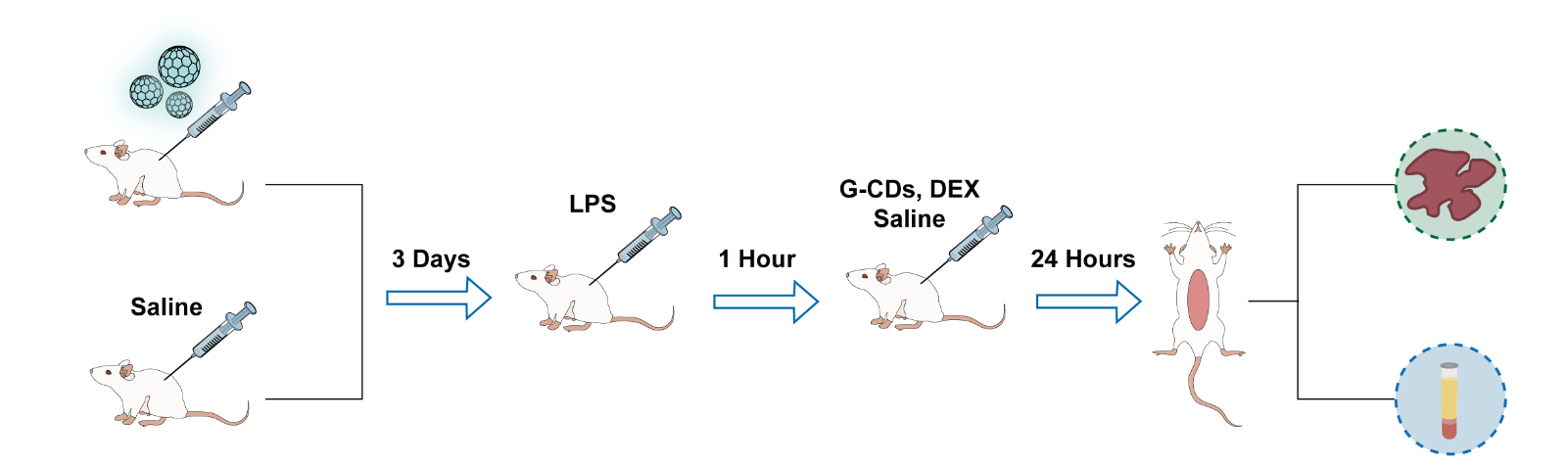


**Figure S9.** Experimental design of the septic liver injury mouse model.


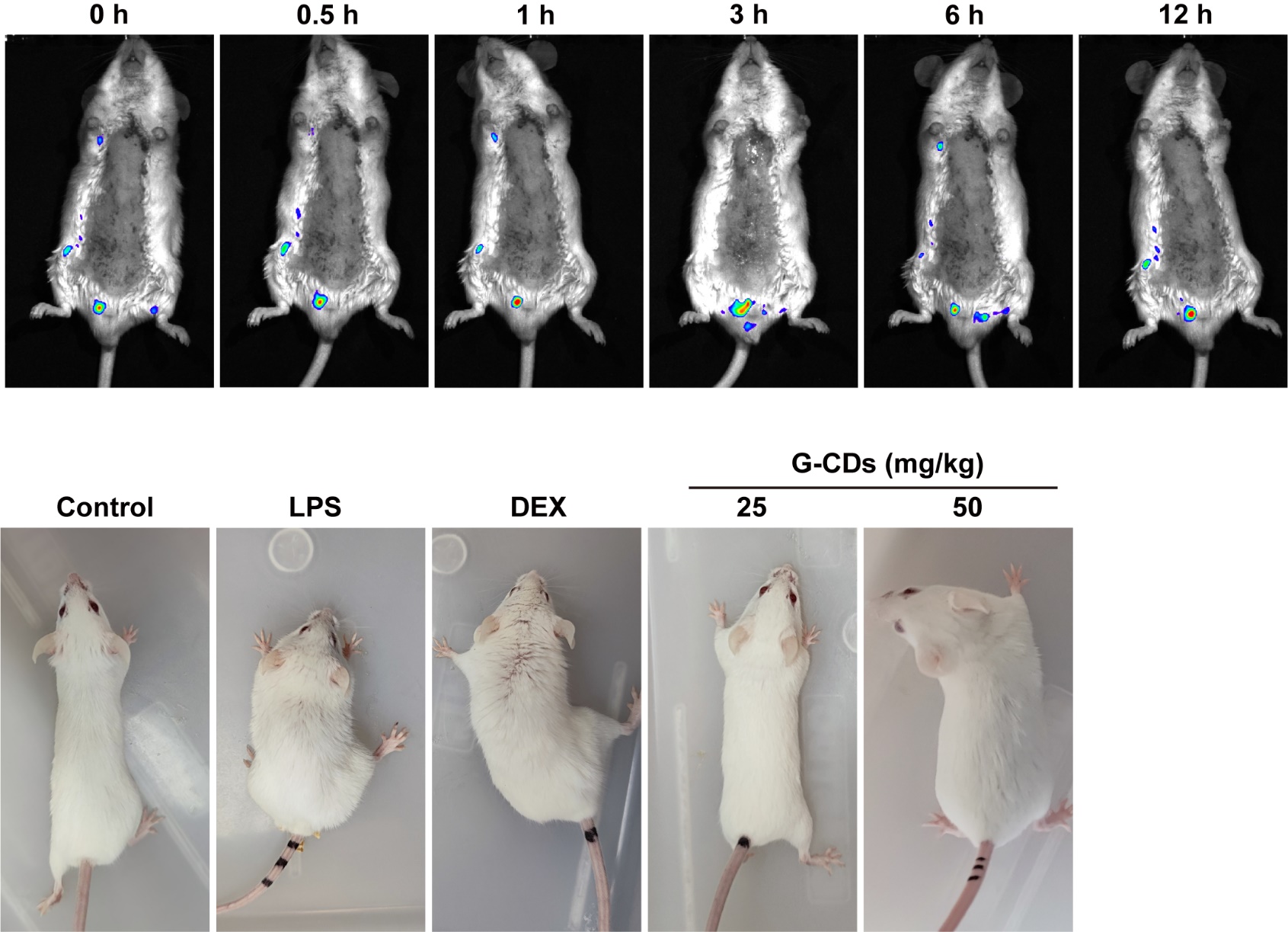


**Figure S10.** The representative images of each group of mice in SILI.


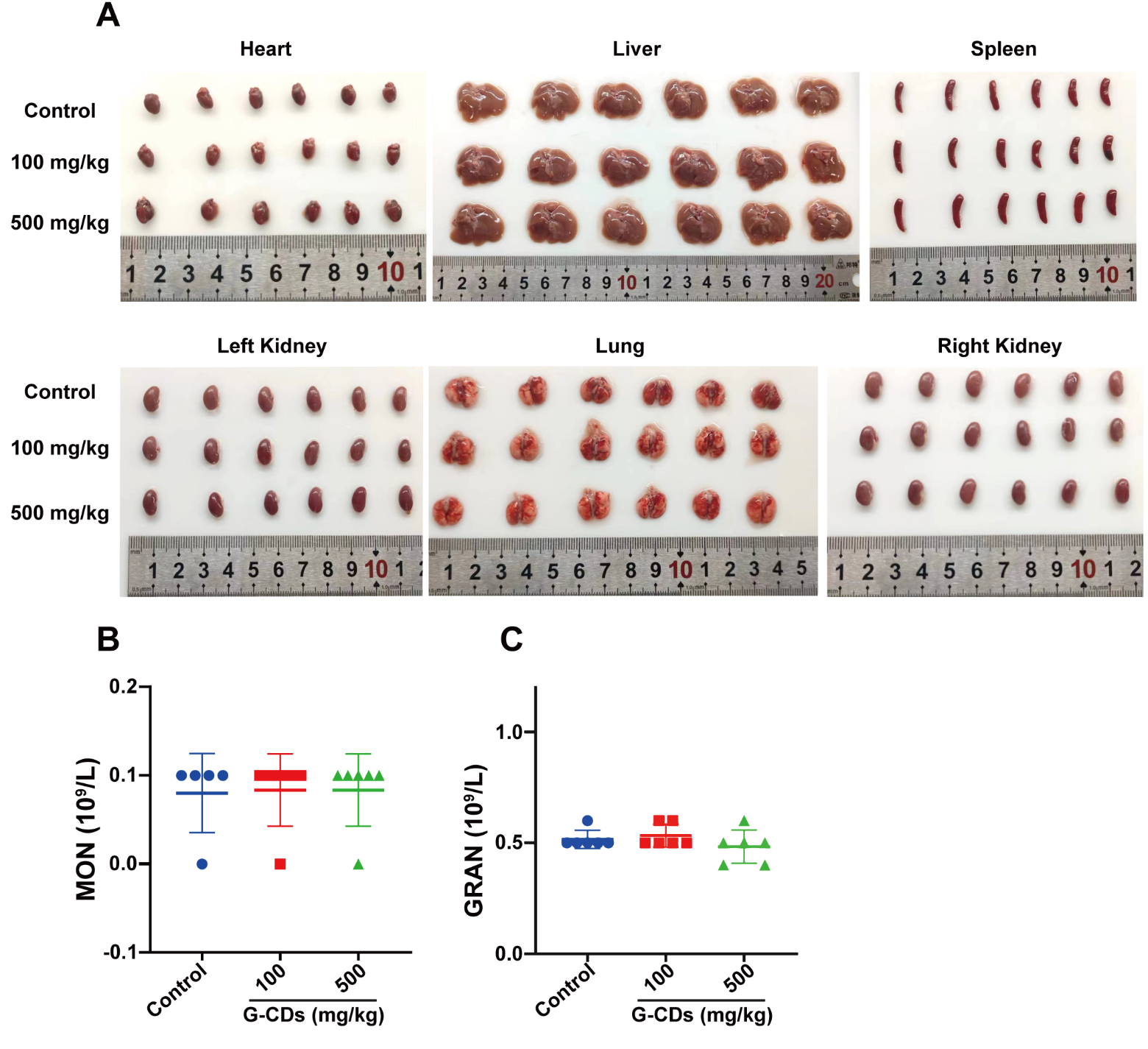


**Figure S11.** (A) Acute toxicity test organography; (B and C) peripheral blood MON and GRAN levels, n=6.


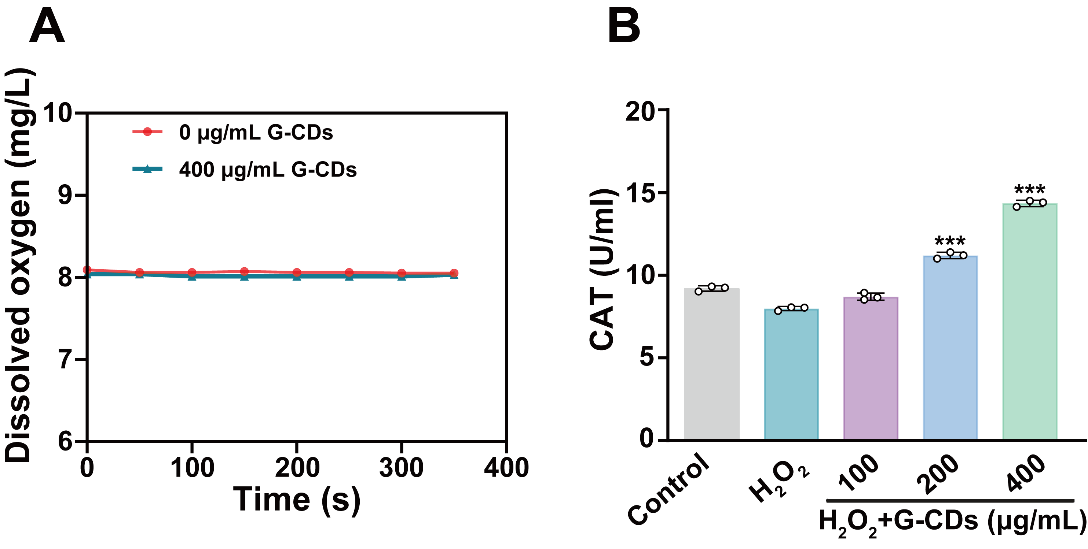


**Figure S12.** (A) Evaluation of the activity of G-CDs in catalyzing the decomposition of H_2_O_2_; (B) Quantitative analysis of CAT activity in RAW264.7 cells. Data are expressed as mean ± SD. *^###^P<0.001*, vs. control group*; *P<0.05, **P<0.01, ***P<0.001,* vs. H_2_O_2_ group, NS means not significant, n=3.


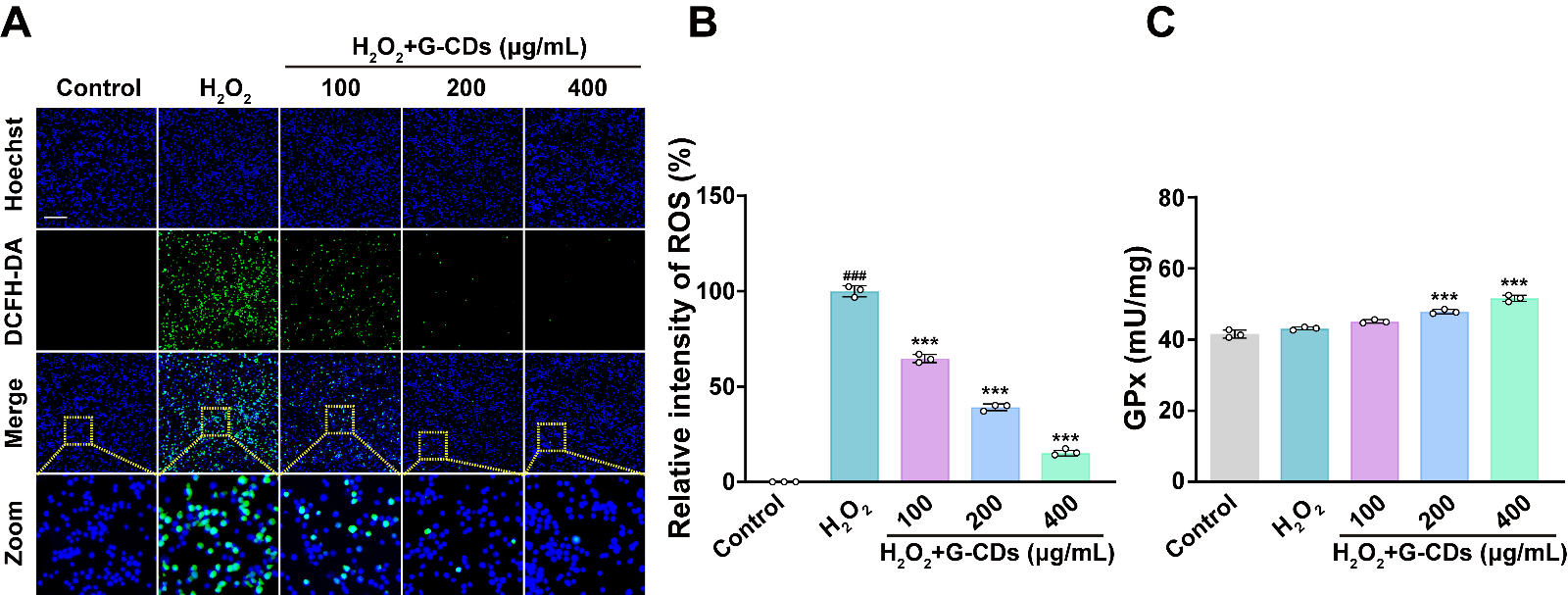


**Figure S13.** (A, B) Detection of ROS using DCFH-DA and Hoechst staining, with quantification of representative fluorescence images (scale bar: 200 μm); (C) Quantitative analysis of GPx activity in RAW264.7 cells. Data are expressed as mean ± SD. *^###^P<0.001*, vs. control group*; ***P<0.001,* vs. H_2_O_2_ group, n=3.


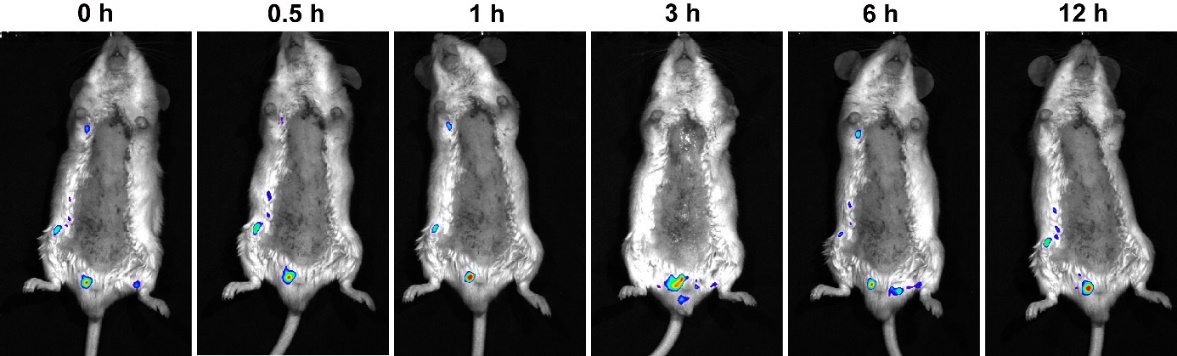


**Figure S14.** Images of in vivo imaging of mice

**Supplementary Table**

**Table S1. Primer sequences used in this study.**

| Gene | Sence (5′ - 3′) | Anti-sense (5′ - 3′) |
| --- | --- | --- |
| IL-6 | TGATGCACTTGCAGAAAACA | ACCAGAGGAAATTTTCAATAGGC |
| IL-1β | GCAACTGTTCCTGAACTCAACT | ATCTTTTGGGGTCCGTCAACT |
| TNF-α | CCCTCACACTCAGATCATCTTCT | GCTACGACGTGGGCTACAG |
| Arg-1 | GGTTCTGGGAGGCCTATCTT | CACCTCCTCTGCTGTCTTCC |
| CD206 | GAGGGAAGCGAGAGATTATGGA | GCCTGATGCCAGGTTAAAGCA |
| 18s | GCAATTATTCCCCATGAACG | GGCCTCACTAAACCATCCAA |
